# Digital imaging of root traits (DIRT): a high-throughput computing and collaboration platform for field-based root phenomics

**DOI:** 10.1186/s13007-015-0093-3

**Published:** 2015-11-02

**Authors:** Abhiram Das, Hannah Schneider, James Burridge, Ana Karine Martinez Ascanio, Tobias Wojciechowski, Christopher N. Topp, Jonathan P. Lynch, Joshua S. Weitz, Alexander Bucksch

**Affiliations:** School of Biology, Georgia Institute of Technology, Atlanta, GA USA; Department of Plant Science, Pennsylvania State University, State College, PA USA; School of Agriculture and Veterinary Medicine, University of Bologna, Bologna, Italy; Forschungszentrum Jülich IBG-2 Pflanzenwissenschaften, Jülich, Germany; Donald Danforth Plant Science Center, St. Louis, MO USA; School of Physics, Georgia Institute of Technology, Atlanta, GA USA; School of Interactive Computing, Georgia Institute of Technology, Atlanta, GA USA

## Abstract

**Background:**

Plant root systems are key drivers of plant function and yield. They are also under-explored targets to meet global food and energy demands. Many new technologies have been developed to characterize crop root system architecture (CRSA). These technologies have the potential to accelerate the progress in understanding the genetic control and environmental response of CRSA. Putting this potential into practice requires new methods and algorithms to analyze CRSA in digital images. Most prior approaches have solely focused on the estimation of root traits from images, yet no integrated platform exists that allows easy and intuitive access to trait extraction and analysis methods from images combined with storage solutions linked to metadata. Automated high-throughput phenotyping methods are increasingly used in laboratory-based efforts to link plant genotype with phenotype, whereas similar field-based studies remain predominantly manual low-throughput.

**Description:**

Here, we present an open-source phenomics platform “DIRT”, as a means to integrate scalable supercomputing architectures into field experiments and analysis pipelines. DIRT is an online platform that enables researchers to store images of plant roots, measure dicot and monocot root traits under field conditions, and share data and results within collaborative teams and the broader community. The DIRT platform seamlessly connects end-users with large-scale compute “commons” enabling the estimation and analysis of root phenotypes from field experiments of unprecedented size.

**Conclusion:**

DIRT is an automated high-throughput computing and collaboration platform for field based crop root phenomics. The platform is accessible at http://dirt.iplantcollaborative.org/ and hosted on the iPlant cyber-infrastructure using high-throughput grid computing resources of the Texas Advanced Computing Center (TACC). DIRT is a high volume central depository and high-throughput RSA trait computation platform for plant scientists working on crop roots. It enables scientists to store, manage and share crop root images with metadata and compute RSA traits from thousands of images in parallel. It makes high-throughput RSA trait computation available to the community with just a few button clicks. As such it enables plant scientists to spend more time on science rather than on technology. All stored and computed data is easily accessible to the public and broader scientific community. We hope that easy data accessibility will attract new tool developers and spur creative data usage that may even be applied to other fields of science.

**Electronic supplementary material:**

The online version of this article (doi:10.1186/s13007-015-0093-3) contains supplementary material, which is available to authorized users.

## Background

Global food demand is projected to double by the year 2050 [1, 2]. Meeting this increased demand requires significant improvements in crop yield and the development of crop plants adapted to water-stress [3] and low fertility soils [4, 5]. Breeding more efficient roots is increasingly recognized as a high-priority target to achieve yield improvements [6] because roots are essential for nutrient and water uptake [7–9]. Yet, little is known regarding the relationship between root system architecture (RSA) and crop function with few examples linking root phenotype with genotype and phenotypic advantages under given field conditions [10–12].

Developing new crop varieties includes both laboratory- and field-based studies [13, 14]. Especially field studies to characterize RSA of mature field-grown crops involve laborious manual tasks that limit the achievable sample size. Extending field-based studies and sample sizes is a widely shared goal for future phenotyping scenarios [15, 16]. Indeed, phenotyping rather than genotyping is recognized as the bottleneck limiting advances [17, 18], given inexpensive next-generation sequencing technologies that have paved the way for characterizing the genotypes of diversity panels of thousands of recombinant inbred lines [19]. In response, a number of national and international efforts, including the International Plant Phenotyping Network, have established “plant phenomics” centers to quantify plant phenotypes and their genetic origin [20].

Similarly, despite some successes, there are relatively few publicly available root phenotyping datasets [21]. Available large datasets are pre-dominantly derived from laboratory-based root phenotyping platforms. Laboratory studies benefit from increased levels of control and, at least in a few cases, have identified loci with candidate genes underlying RSA in early root development [22, 23]. However, growth containers used in these studies, filled with real or artificial soil [24–27], limit observations spatially and temporally to small or immature root systems [28, 29].

Establishing a link between RSA and genotypes requires the measurement of root phenotypes [30], often derived from automatic analysis of two-dimensional and three-dimensional digital images [31–39]. A comprehensive overview of existing software for root image analysis is maintained at the site: http://plant-image-analysis.org [40]. The scope of this software collection is impressive, in that individual tools provide different degrees of computational automation, ranging from manual, semi-automatic to fully automatic. However, none of these provide an integrated platform that can (a) associate root images with environmental and phenotypic meta-data, (b) provide seamless access to scalable, supercomputing resources for non-technical users and (c) share information within a collaborative team and the plant science community.

In order to address these issues we have developed DIRT. The DIRT platform provides a number of major functionalities that enable researchers to: (a) manage root image collections and metadata; (b) interactively calibrate measurement pipelines; (c) compute crop root traits on scalable high-throughput compute platforms; and (d) analyze the results of computations. Broadly DIRT enables researchers to process thousands of root images through the pipeline with custom parameters and view and analyze computed RSA output associated to the raw images. Thus, our platform makes high-throughput scalable computational platforms available to the researchers with no technical expertise.

## Utility

**Fig. 1:**
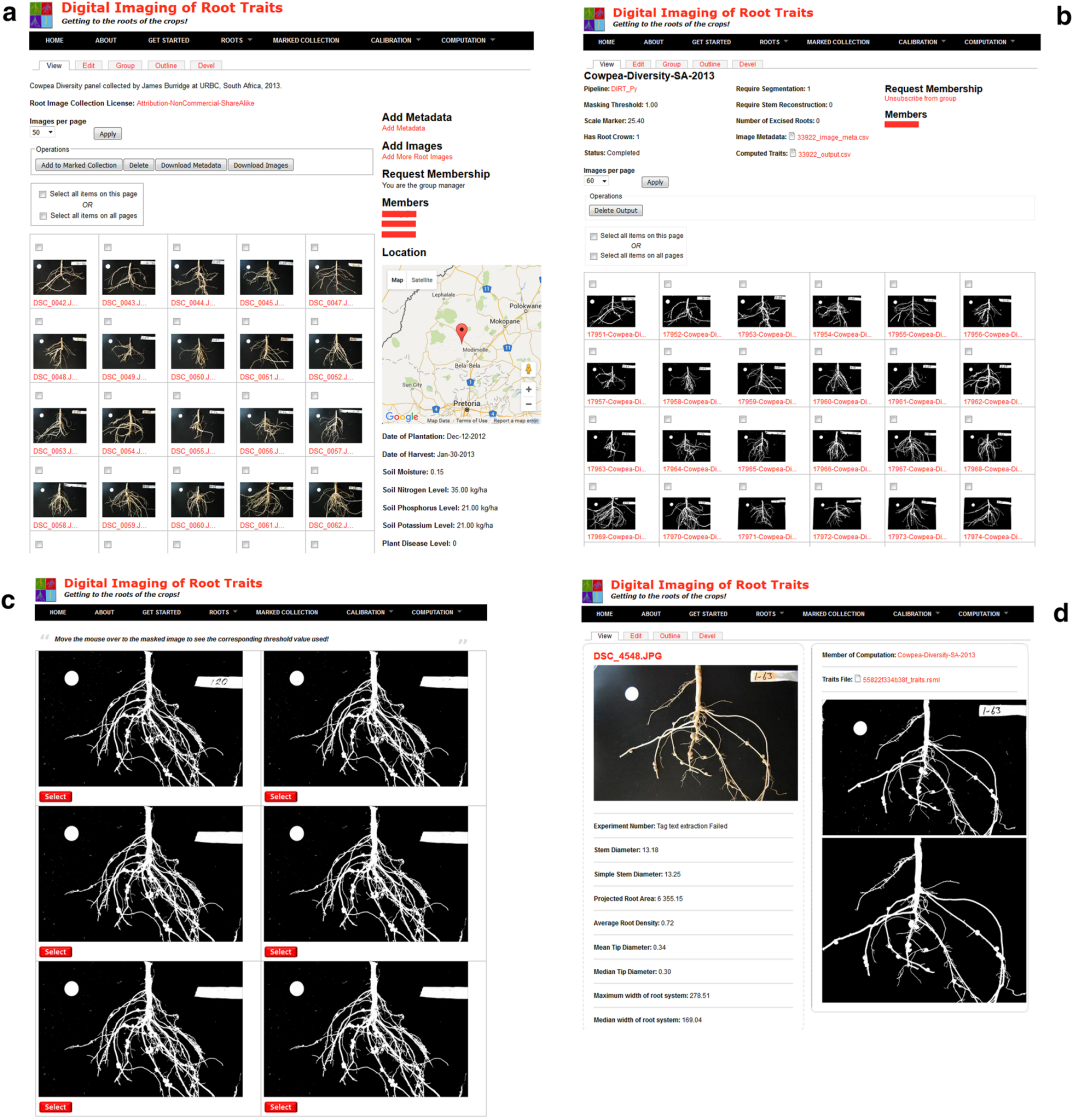
Major DIRT functionalities. **a** A cowpea root dataset annotated with experiment parameters and location and shared with three other members (names were replace with *red bars*). **b** The overview of the computed cowpea data set shown in (**a**). The computation parameters are shown along with *icons* of the image mask. Computed traits and entered image metadata can be downloaded as Excel compatible.csv files. **c** A user can visually choose the best threshold parameter to separate the root from the background. **d** Each of the images in the computation shown in (**b**) can be assessed in detail. Every image processing step can be followed visually per image and compared to the original image and the computed traits

DIRT addresses the phenotyping bottleneck within the computational plant sciences, by providing a single platform to meet the demands of data access and storage, exchange and sharing, and image-based high-throughput root phenotyping [41]. The DIRT platform enables users to organize and share images as datasets per experiment (Fig. 1a), run image processing algorithms on the datasets such that computed root trait values can be downloaded directly from the user interface (Fig. 1b). Visual quality control is implemented as a calibration tool for the masking threshold needed to separate the root from the background (Fig. 1c) and the possibility to investigate all intermediate image processing steps (Fig. 1d). The algorithms deployed on DIRT have been specifically designed and tested on two-dimensional images taken of root systems in the field. By focusing on crop root traits, DIRT also overcomes the time consuming manual measurement processes involved in Shovelomics [42], while enabling measurements of manually inaccessible traits such as the dominant root tissue angle. Overall, DIRT is a unique root phenotyping platform, accessible by everybody via an interactive web-based interface without the need to install software locally on a computer.

The RSA trait computation pipeline available in DIRT is fully automated and includes automatic estimation of 78 traits in total (see Additional file 1: Section S3). Traits are categorized into common traits for all root system architectures, monocot traits, dicot traits and traits for excised root samples. We provide a separate, optional threshold calibration tool that allows the researcher to select a representative image from the marked collection and compute binary image masks using different segmentation threshold values. Within this calibration workflow, the user selects the most appropriate value by visually checking the image mask.

As a response to community requests, the original trait computation pipeline in DIRT was extended. The current pipeline includes previously unpublished algorithms to measure traits such as top and bottom angle in monocots (see Additional file 1: Section S3). The pipeline is best used by following the DIRT imaging protocol to process 2D root images. In brief, a washed root is imaged against a dark diffuse reflecting background that contains a light colored circle with known diameter. Additionally, a barcode, QR-code or simple text can be placed above the root for automatic identification to be associated with trait computations (see Additional file 1: Figure S2). On completion of the computation, masked images, computed traits, and corresponding CSV and RSML files [43] populate the computation view tab. See Additional files 2 and 3 for examples of produced CSV and RSML files.

DIRT was designed to enable full data control for researchers, whether individually or as part of collaborative teams. As such we realized sharing options, where each newly created collection is designated to be private by default. The owner of a collection can share data and computed results privately with one or many collaborators via the platform’s web-interface or publish collections and computations publically under a chosen creative commons license. Furthermore, DIRT enables different functions based on user access rights. The owners of data can edit, upload, download and delete images and corresponding metadata. Metadata can be associated to whole experiments or data sets to document experiment conditions (e.g. FAO soil type, GPS location, soil moisture content). The association is realized as an upload of a CSV file containing the metadata or is entered via a web form directly in the web browser. On top of suggested standard experiment parameters a dynamic form allows the documentation of non-standard parameters such as nitrogen content per depth level. Similarly, each root image can be annotated manually or by uploading a pre-formatted CSV file with specific metadata (e.g. genotype, dry biomass) and may contain RSML files of manual measurements to annotate the image, e.g. from RootNav [44] (Additional file 1: Section S6.3.7).

DIRT is hosted publically on the iPlant cyber-infrastructure [45, 46] leveraging its cloud data storage and the Advanced Agave API to communicate with the Texas Advanced Computing Center (TACC) for high-throughput computation of stored root images. It is built as a multi-tiered application consisting of a web server, a database server, iPlant’s data store, middleware and grid computing. The core middleware components are the PHP modules interfacing the database, iPlant data store and grid-computing environment. DIRT’s web interface is developed using the widely adopted open source content management system Drupal (http://drupal.org). DIRTs’ graphical interfaces (Fig. 1) are accessible via standard web browsers and abstract the organization and storage of root images and their metadata in a MySQL database and iPlant’s data store from the user. The image-processing pipeline is developed in Python and runs on TACC. The trait computation pipeline is abstracted from the computational resources and from the aggregation and sharing of images. Hence, it is possible for developers to extend DIRT by incorporating new pipelines adapted to distinct imaging and experiment conditions (see Additional file 1: Section S7.3). The DIRT source code and installation instructions are available for download from the DIRT website (see Additional file 1: Section S7.2) to facilitate use of private supercomputing resources for the plant science community. As a proof of concept we have also released an installation of DIRT at Georgia Tech (http://dirt.biology.gatech.edu) that uses Georgia Tech’s high performance computing environment; instructions for a local installation of DIRT on proprietary computing resources are described in Additional file 1: Section S7.3. Altogether, DIRT assembles a unique root phenotyping platform that is accessible to non-technical users via an interactive web-based interface.

## Design and implementation

**Fig. 2:**
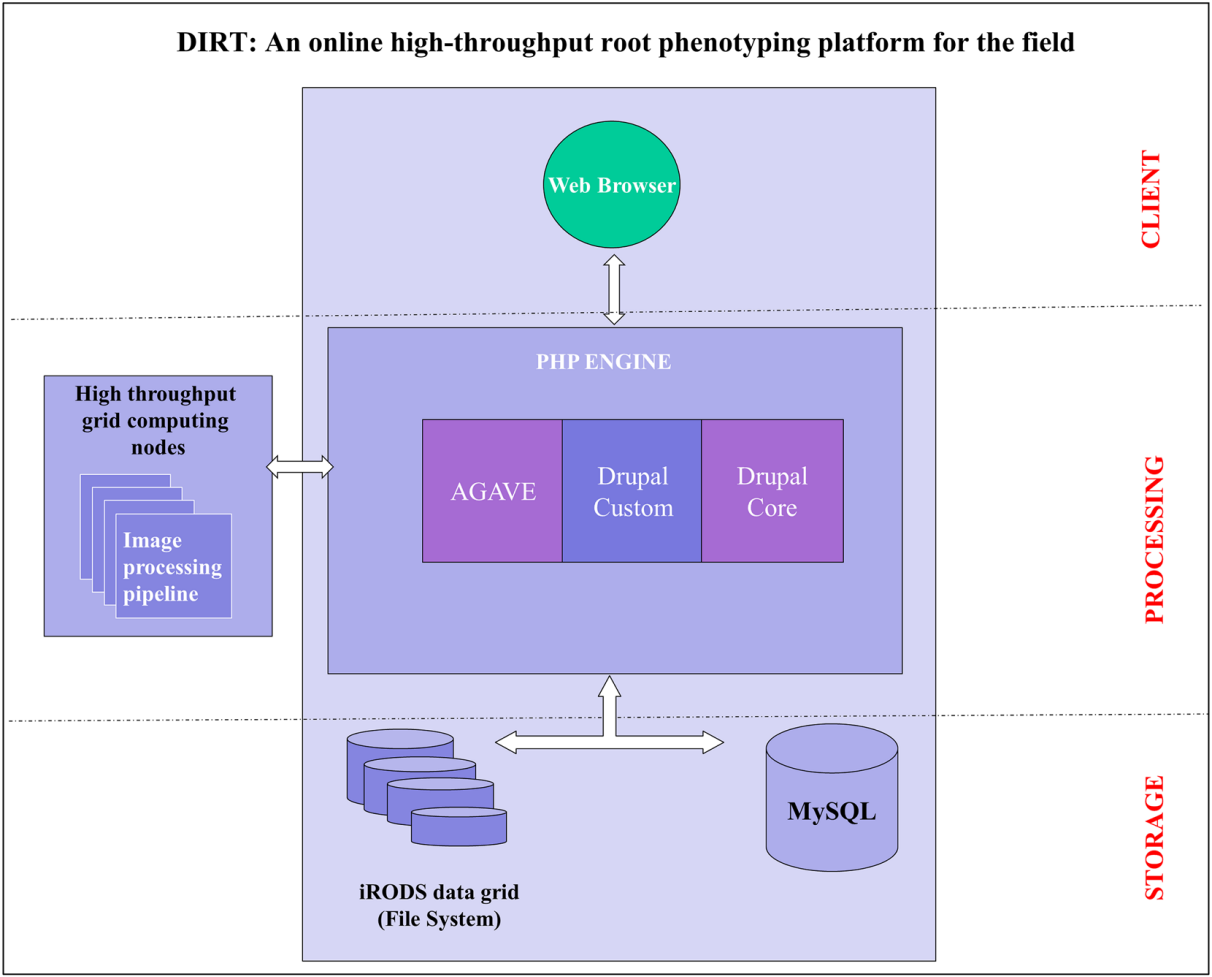
DIRT Architecture. DIRT is programed within the framework of the Drupal content management system and can be configured to interact with any high-throughput grid computing environment. The iPlant installation uses the Agave API to communicate with the high-throughput computing environment. The Agave API is utilized to transfer images from the iPlant data store to the computing platform and execute the computation. Metadata is organized in a MySQL database. All customized Drupal modules are open source and provide the functionality for configuration and communication with remote high-throughput grid computing platform

In this section we describe the high-level view of the system to give insight into the extensibility and sustainability rationale underlying the platform design. DIRT is a multi-tiered online platform developed with the Drupal framework [47]. Drupal is an open source content management system and framework made up of a software stack that can be used to build content-rich web applications. Figure 2 shows the three tier architecture: the client tier constitutes the user interfaces in web browser, the processing tier encompasses the Drupal modules and image processing pipeline, and the storage tier consists of the database and file systems. Figure 1 shows a high-level overview of the interfaces available to DIRT users. In particular, the functional specifications of DIRT were defined to meet the demands of field root phenotyping:

1. Private and public storage of large root image data sets with metadata for each image and data set. In doing so, DIRT users don't have to be concerned with the computational and storage needs.
2. The platform supports private virtual collections by selecting root images from different physical collections. Virtual collections have the potential to save time and money required for new field experiments, by simply combining existing experiments.
3. Up-scaling of RSA trait estimation to supercomputing platforms.
4. The DIRT platform should allow storage of different types of image data.
5. The DIRT platform is extensible to incorporate new RSA trait computation pipelines.
6. The source code of the DIRT platform is freely available under open source licenses to the science community.

To meet the above platforms design specifications, we chose the following software stack to develop and build the application:

1. For the RSA trait estimation we chose the pipeline developed in Python (see Additional file 1: Section S3) and ported it to grid computing infrastructure for high-throughput computation.
2. User interfaces, user management, access control, data management, application workflow, user task scheduling and system’s configuration were implemented as open-source Drupal modules.
3. The public DIRT installation on iPlant interfaces with the STAMPEDE high performance computing platform at TACC [48].
4. For scalable storage and public infrastructure we chose the data store within iPlant’s cyber-infrastructure.
5. The communication between DIRT and STAMPEDE is realized with the AGAVE API [49] and a secure shell connection.

In the following we detail the content, component and deployment model of the DIRT system to inform developers about our extensions to DRUPAL.

### Content model

**Fig. 3:**
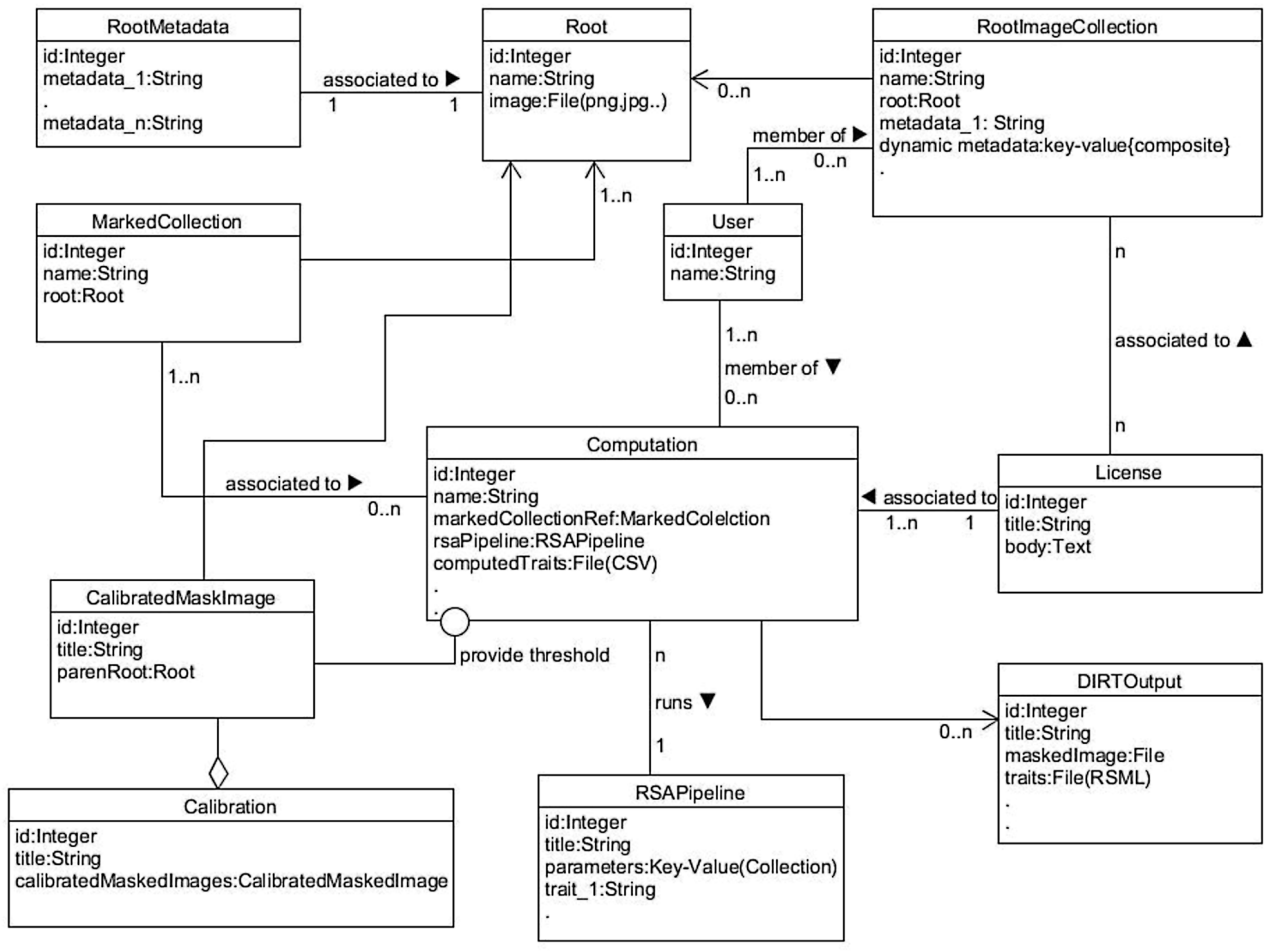
Content model or class association diagram of DIRT. A class association diagram in UML [50] is a static structure diagram to describe the relationship between the contents or classes of a software system including their class attributes. This figure shows the custom contents or classes, their attributes and relationship within DIRT. The *rectangular boxes* represent the content, *the text on top portion* of the box represents the name of the content and *the text in lower portion* of the box represents important content attributes. The *line* connecting the boxes denote the content association. The *symbols* at the end of these lines represent the association type and the text on these lines represents the attributes of the association

The content model is best described as a class association model that defines the storage architecture for contents with different attributes (e.g. root images, collections, virtual collections, metadata). A class association diagram in the unified modeling language (UML) [50] is a type of static structure diagram that describes the structure of a system by showing the system’s contents or classes, their attributes, operations and relationships. Figure 3 is a class association diagram of the DIRT’s contents or classes depicting the major attributes or fields of the contents and their relationships.

Within the Drupal framework, each content type has a set of common attributes:

- NID (Node ID): Every node or content in the Drupal system has a unique ID assigned, irrespective of the content type.
- Title: Every node or content in the system is required to have a title.
- UID: Every node or content in the system is explicitly tied to its creator i.e. the user of the system who created it.
- Status: Every node or content in the system has one of the two states, published or unpublished. This feature assures that content is kept offline, until the content is valid and complete to be taken online.
- Created and changed: A timestamp monitor content or node changes.
- VID (version ID): Every node or content in the system maintains its version information. If enabled, all changes to a content or node is stored and maintained.

In addition to Drupal’s common attributes, DIRT content types require custom attributes to meet the system’s requirement specifications. Here we describe these content types briefly:

- Calibrated mask images contains attributes to associate an image to multiple image masks created during the calibration of an original root image and an attribute referencing the original root image in the system database.
- Computation references to a marked collection, a RSA trait computation pipeline, the pipeline parameters and the traits available in a pipeline. Furthermore, Computation contains an attribute to define its visibility. A computation also contains a field of type *file* to link to a CSV file containing computed RSA trait values of a referred Marked Collection.
- DIRT Output defines the output produced for each raw root image by the RSA trait computation pipeline. It contains attributes to refer to a computation and original root image. Additionally, the content type contains attributes to refer to the image mask of the original image, each RSA trait value and the output RSML file.
- Image processing pipeline has attributes for the pipeline parameters and each available trait.
- License defines attributes for the licenses supported by the DIRT platform. The License content type is associated to computation and root image collection content types.
- Marked collection has attributes that describe a list of root images.
- Metadata has attributes that refer to a root image collection and a file that links a pre-formatted CSV file.
- Root refers to an original root image within a root image collection. Hence, the attributes hold a reference to a root image, a root image collection and each associated metadata entry.
- Root image collection has the attributes collection visibility, collection, membership, collection license and all collection metadata.

### Component model

The DIRT platform consists of three major components:

1. Web server component: These are the Drupal components including core, community contributed and custom DIRT modules that orchestrate the whole platform in cohort. The content model described in the previous section is designed and implemented using these module types.
2. RSA trait computation component: These are the Python code used for the trait computation that is deployed to both the web server and grid computing node to meet the calibration and trait computation system specifications respectively.
3. Interface component: These are the shell scripts that reside on the web server and grid-computing node to interface between DIRT and the grid job scheduler.

**Fig. 4:**
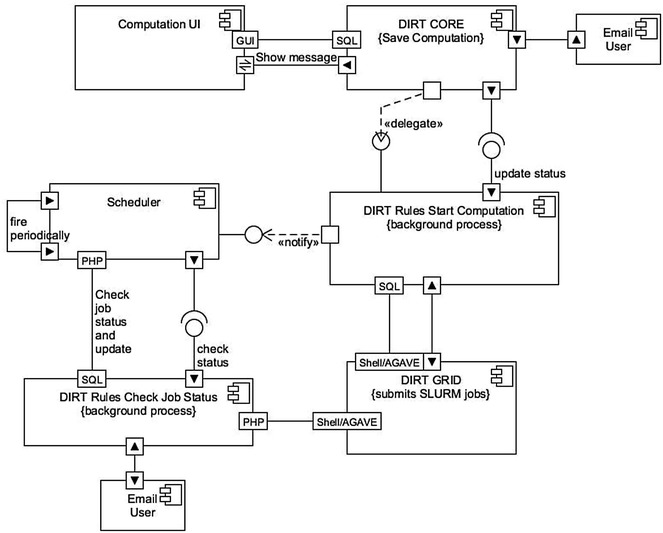
Component diagram showing the components involved in RSA trait computation process on the DIRT platform. In UML [50], a component diagram represents the structural relationships between the components that form larger subsystems. A component is considered as an autonomous, encapsulated unit within a software system that provides one or more interfaces

In accordance with the Drupal architecture guidelines, DIRT is modular and every process in DIRT involves several components or modules. In Fig. 4 we show the components and their interactions in DIRT for the RSA trait computation process. The computation process in DIRT involves the user interface component, rules component, workflow component, custom DIRT components and core components. The processes start whenever a new content of type “Computation” is created. The user provides a computation name, selects a “Marked Collection” and the RSA trait computation pipeline, provides the respective pipeline parameters and selects traits to be computed. By clicking the “Save” button in the computation interface the rules engine is notified to trigger two DIRT workflows. The first workflow starts the DIRT job submission module as background process to run the RSA pipeline on the grid-computing environment. The background process receives the configuration details of the grid job, updates the database system, changes the computation status and notifies the user about the computation status via email if the computation is started successfully. As a second workflow the background process schedules the DIRT job status check module to run in background in every 10 min (until job completion or termination). When executed the grid is pinged for the job status and the job status is checked. If the job is completed, the computed output is transferred to the web server, the database is updated with the computed values, DIRT output contents are created and the user is notified. Each step in these workflows in turn is associated with other sub-modules or components located across different software nodes of the platform.

### Deployment model

The deployment model is the static view of the run-time configuration of the processing nodes and all executed components. The deployment model defines the distribution of all DIRT components across different physical nodes in terms of folder structures an access rights. This deployment model is largely automated. Therefore we refer for detailed practical information to the Additional file 1: Section S7.

## Discussion and conclusion

**Fig. 5:**
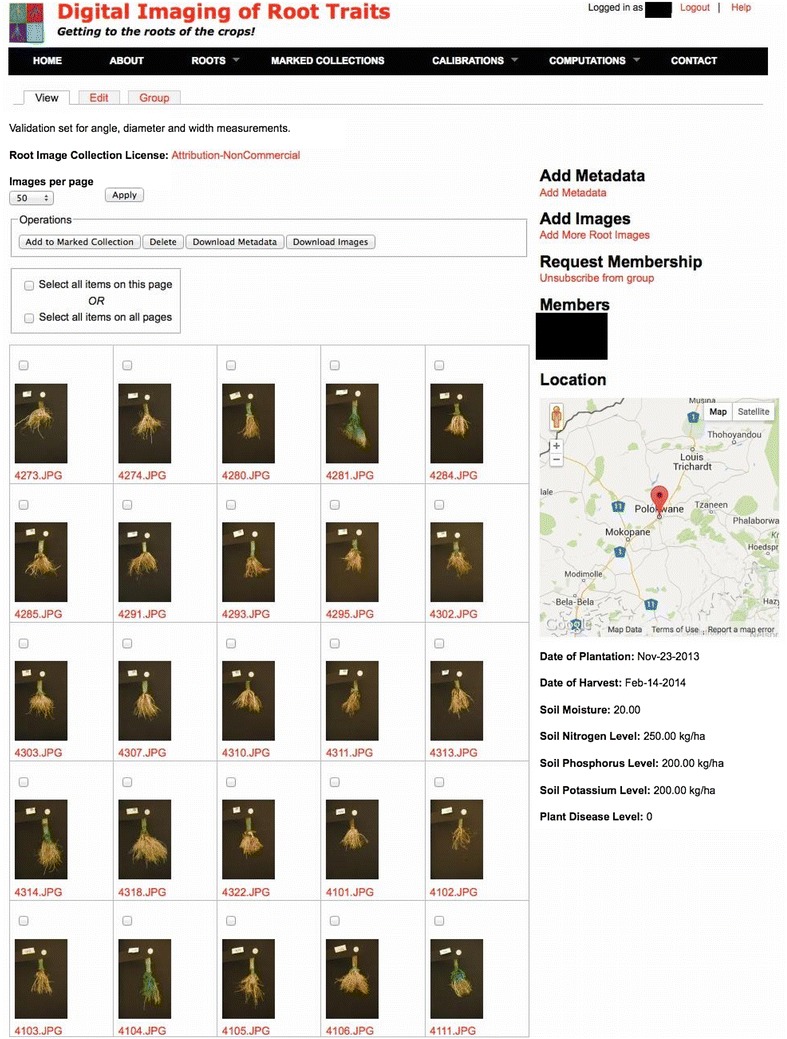
Screenshot from the DIRT web-application. The screenshot shows the root collection overview tab for a maize validation data set collected at the Ukulima Root Biology Center in South Africa. On the top the main menu is visible that contains all functionality to manage root images, create marked collections, run computations and perform the threshold calibration. Individual root images are shown below, along with an informal description of the dataset, an accompanied creative commons license and the location of the root excavation

**Fig. 6:**
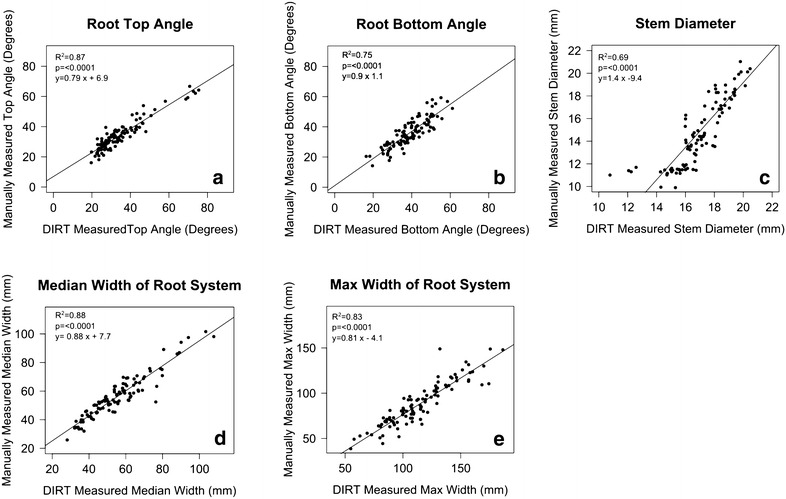
Validation of DIRT traits. **a** root top angle, **b** root bottom angle, **c** stem diameter, **d** median width of the root system and **e** maximum width of the root system for the public maize data set accessible at http://dirt.iplantcollaborative.org/content/maize-validation-set. The data set contains 99 maize roots

DIRT is designed as a community platform. As such we collected 10 public data sets that are available to every iPlant user. These initial data sets contain 4894 root images of field-grown roots excavated with the shovelomics technique. Four of these data sets are published on DIRT before the publication of their related projects. Furthermore, we expect the content volume to grow rapidly through additions from the plant science community. This expectation relies on the observed growth of DIRT users. At time of publication we counted 31 users from 14 institutions and we are confident that our users follow the open science example of open data, source code and documentation sharing. For example, in Figs. 5 and 6 we show a typical community contribution, where a public maize dataset (Fig. 5) is used to compare and validate the manual Shovelomics traits with automatically computed DIRT traits (Fig. 6). In the given maize example, previously unavailable traits were added to DIRT (Root Top Angle, Root Bottom Angle) and subsequently validated by the DIRT user community. The data set and its stored computation results were shared on the website (http://dirt.iplantcollaborative.org/content/maize-validation-set) along with a reference to the validation presented in this paper. Overall, the contributed validation showed excellent results by reassuring the known correlations of stem diameter (R^2^ = 0.69, p < 0.0001), median width (R^2^ = 0.88, p < 0.0001) and maximum width (R^2^ = 0.83, p < 0.0001), as well as establishing new correlations for the previously unpublished traits root top angle (R^2^ = 0.87, p < 0.0001) and root bottom angle (R^2^ = 0.75, p < 0.0001). Details on other public data sets can be found in the Additional file 1: Section S5.

From our experience, the simple excavation and imaging protocol enables 2–3 persons to phenotype 500–700 common bean roots per day in soil with high clay content. Here, the limiting factor are soil properties such as clay content or compactness that impede root excavation, while sandy soils allow fast and easy root excavation. Until now we did not experience the limits of the computing resources. However, the growing community of DIRT users will increase the computational load on the computing resources and eventually reveal the limits of the current system.

We presented DIRT as an open online platform that stores and organizes root image data sets, executes RSA trait estimations and documents performed computations on root image data sets. DIRT allows contributions from the whole root phenotyping community, including users and developers, and enables sharing and documentation of experiments. It is encouraged to submit images taken with the DIRT imaging protocol to make use of all DIRT features. However, proprietary imaging protocols are often supported with limitations. Additionally, our efforts to make DIRT an open-source, transparent and freely accessible tool will enable further development and adaptation of the platform in response to research demands of free public data sets [21]. Overall DIRT is a unique computational resource that promotes automated, yet researcher independent, root phenotyping as a response to the demands of researchers working under field conditions, to discover novel links between root morphology and the plant genome.

## Availability and requirements

DIRT is freely accessible and usable at http://dirt.iplantcollaborative.org. In the spirit of open-source development, we have hosted DIRT on iPlant’s cyber infrastructure, which is open to the public. All source code is available on the DIRT GitHub repository (https://github.com/abucksch/DIRT) and on the DIRT website (http://dirt.iplantcollaborative.org/about-us?qt-about_us_quicktabs=2#qt-about_us_quicktabs). A user manual guide is included as part of the Additional file 1.

## Additional file

10.1186/s13007-015-0093-3 Detailed technical information about the DIRT platform including a quickstart guide for users and a installation guide for developers.
10.1186/s13007-015-0093-3 Example of the DIRT output formating.
10.1186/s13007-015-0093-3 Example of the RSML output provided by the DIRT platform.

## References

[1] Godfray HCJ (2010). Food security: the challenge of feeding 9 billion people. Science.

[2] Tilman D (2011). Global food demand and the sustainable intensification of agriculture. Proc Natl Acad Sci.

[3] OECD. OECD Environmental Outlook to 2030. OECD Publishing; 2008.

[4] Lynch JP (2007). Roots of the second green revolution. Aust J Bot.

[5] López-Arredondo D, González-Morales SI, Bello-Bello E, et al. Engineering food crops to grow in harsh environments [version 1; referees: 2 approved]. F1000Research 2015, 4(F1000 Faculty Rev):651. doi:10.12688/f1000research.6538.1. 10.12688/f1000research.6538.1 PMC4560252 26380074

[6] Araus JL, Cairns JE (2014). Field high-throughput phenotyping: the new crop breeding frontier. Trend Plant Sci.

[7] Beebe SE (2006). Quantitative trait loci for root architecture traits correlated with phosphorus acquisition in common bean. Crop Sci.

[8] Saengwilai P, Tian XL, Lynch JP (2014). Low crown root number enhances nitrogen acquisition from low-nitrogen soils in maize. Plant Physiol.

[9] Waisel Y (2002). Plant roots: the hidden half. Ann Bot.

[10] de Sousa SM (2012). A role for root morphology and related candidate genes in P acquisition efficiency in maize. Funct Plant Biol.

[11] Lynch J (1995). Root architecture and plant productivity. Plant Physiol.

[12] Zhu JM (2011). From lab to field, new approaches to phenotyping root system architecture. Curr Opin Plant Biol.

[13] Jansen M (2014). Non-invasive phenotyping methodologies enable the accurate characterization of growth and performance of shoots and roots. Genomics of plant genetic resources.

[14] Rogers ED, Benfey PN (2015). Regulation of plant root system architecture: implications for crop advancement. Curr Opin Biotechnol.

[15] Fiorani F, Schurr U (2013). Future scenarios for plant phenotyping. Annu Rev Plant Biol.

[16] Wuyts N, Dhondt S, Inzé D (2015). Measurement of plant growth in view of an integrative analysis of regulatory networks. Curr Opin Plant Biol.

[17] Kuijken RCP (2015). Root phenotyping: from component trait in the lab to breeding. J Exp Bot.

[18] Rahman H (2015). Phenomics: technologies and applications in plant and agriculture. PlantOmics: the omics of plant science.

[19] McMullen MD (2009). Genetic properties of the maize nested association mapping population. Science.

[20] Finkel E (2009). IMAGING with ‘Phenomics’, plant scientists hope to shift breeding into overdrive. Science.

[21] Fahlgren N, Gehan MA, Baxter I (2015). Lights, camera, action: high-throughput plant phenotyping is ready for a close-up. Curr Opin Plant Biol.

[22] Topp CN (2013). 3D phenotyping and quantitative trait locus mapping identify core regions of the rice genome controlling root architecture. Proc Natl Acad Sci USA.

[23] Pace J, Yu X, Lübberstedt T (2015). Genomic prediction of seedling root length in maize (Zea mays L.). Plant J.

[24] Clark RT (2011). Three-dimensional root phenotyping with a novel imaging and software platform. Plant Physiol.

[25] Downie H (2012). Transparent soil for imaging the rhizosphere. PLoS One.

[26] Iyer-Pascuzzi AS (2010). Imaging and analysis platform for automatic phenotyping and trait ranking of plant root systems. Plant Physiol.

[27] Rellan-Alvarez R (2015). GLO-Roots: an imaging platform enabling multidimensional characterization of soil-grown root systems. Elife.

[28] Judd LA, Jackson BE, Fonteno WC (2015). Advancements in root growth measurement technologies and observation capabilities for container-grown plants. Plants.

[29] Pfeifer J (2015). Rapid phenotyping of crop root systems in undisturbed field soils using X-ray computed tomography. Plant methods.

[30] Walter A, Liebisch F, Hund A (2015). Plant phenotyping: from bean weighing to image analysis. Plant Method.

[31] Cai J (2015). RootGraph: a graphic optimization tool for automated image analysis of plant roots. J Exp Bot.

[32] Clark RT (2013). High-throughput two-dimensional root system phenotyping platform facilitates genetic analysis of root growth and development. Plant Cell Environ.

[33] Colombi T (2015). Next generation shovelomics: set up a tent and REST. Plant Soil.

[34] Humplik JF (2015). Automated phenotyping of plant shoots using imaging methods for analysis of plant stress responses—a review. Plant Method.

[35] Metzner R (2015). Direct comparison of MRI and X-ray CT technologies for 3D imaging of root systems in soil: potential and challenges for root trait quantification. Plant Method.

[36] Mooney SJ (2012). Developing X-ray computed tomography to non-invasively image 3-D root systems architecture in soil. Plant Soil.

[37] Symonova O, Topp CN, Edelsbrunner H (2015). DynamicRoots: a software platform for the reconstruction and analysis of growing plant roots. Plos ONE.

[38] Yazdanbakhsh N, Fisahn J (2009). High throughput phenotyping of root growth dynamics, lateral root formation, root architecture and root hair development enabled by PlaRoM. Funct Plant Biol.

[39] Delory BM (2015). archiDART: an R package for the automated computation of plant root architectural traits. Plant Soil.

[40] Lobet G, Draye X, Perilleux C (2013). An online database for plant image analysis software tools. Plant Method.

[41] Bucksch A (2014). Image-based high-throughput field phenotyping of crop roots. Plant Physiol.

[42] Trachsel S (2011). Shovelomics: high throughput phenotyping of maize (Zea mays L.) root architecture in the field. Plant Soil.

[43] Lobet G (2015). Root system markup language: toward a unified root architecture description language. Plant Physiol.

[44] Pound MP (2013). RootNav: navigating images of complex root architectures. Plant Physiol.

[45] Goff SA (2011). The iPlant collaborative: cyberinfrastructure for plant biology. Front Plant Sci.

[46] Stanzione D (2011). The iPlant collaborative: cyberinfrastructure to feed the world. Computer.

[47] Drupal. https://drupal.org/ Accessed 16 June 2015.

[48] STAMPEDE at TACC. https://tacc.utexas.edu/systems/stampede Accessed 16 June 2015.

[49] Agave API. http://agaveapi.co. Accessed 16 June 2015.

[50] Fowler M (2004). UML distilled: a brief guide to the standard object modeling language.

