# Supplementary material for: Digital imaging of root traits (DIRT): a high-throughput computing and collaboration platform for field-based root phenomics
Source: Plant Methods. 2015 Nov 2;11:51. doi: 10.1186/s13007-015-0093-3 (PMC4630929; doi:10.1186/s13007-015-0093-3)
Supplement: Supplementary file 1 — 10.1186/s13007-015-0093-3 Detailed technical information about the DIRT platform including a quickstart guide for users and a installation guide for developers. [file 13007_2015_93_MOESM1_ESM.pdf]

Supplementary Material

DIRT: a high-throughput computing and collaboration platform for field-based root phenomics

Abhiram Das[1], Hannah Schneider[2], James Burridge[2], Ana Karine Martinez Ascanio[3], Tobias Wojciechowski[4], Christopher N. Topp[5], Jonathan P. Lynch[2], Joshua S. Weitz[1,6], Alexander Bucksch[1,7,*]

[1]School of Biology, Georgia Institute of Technology, Atlanta, Georgia, USA
[2]Department of Plant Science, Pennsylvania State University, Pennsylvania, USA
[3]School of Agriculture and Veterinary Medicine, University of Bologna, Italy
[4]Forschungszentrum Jülich IBG-2 Pflanzenwissenschaften, Germany
[5]Donald Danforth Plant Science Center,St. Louis, Missouri, USA
[6]School of Physics, Georgia Institute of Technology, Atlanta, Georgia, USA
[7]School of Interactive Computing, Georgia Institute of Technology, Atlanta, Georgia, USA

[*]Corresponding author – Alexander Bucksch (bucksch@gatech.edu)

Contents

1 DIRT Overview 4
2 DIRT pipeline and its parameters 4
3 DIRT RSA traits 5
3.1 Common traits 5
3.2 Dicot root traits 6
3.4 Monocot root traits 7
3.5 Excised root traits 8
4 Output file formats 8
5 Public Data 9
5.1 Image data sets and computations 9
6 Quick start guide 10
6.1 Access DIRT 10
6.2 Login to DIRT 10
6.3 Root image collection 10
6.3.1 Create root image collection 10
6.3.2 Upload images to a collection 10
6.3.3 View root image collections 10
6.3.4 Share root image collection 10
6.3.5 Download images from a collection 11
6.3.6 Download image metadata of a collection 11
6.3.7 Add metadata of images in a collection 12
6.4 Marked image collection 12
6.4.1 Create a marked collection 12
6.4.2 Add images to an existing marked collection 12
6.4.3 View marked collections 12
6.5 Image Threshold Calibration 13
6.5.1 Calibrate threshold value of an image 13
6.5.2 View calibrations 13
6.6 RSA Trait Computation 13
6.6.1 Create a computation 13
6.6.2 View computations 14
6.6.3 Share a computation 14
6.6.4 Cancel a computation 15
6.6.5 Download computed RSA traits 15
6.7 User quota 15
7 Developer guide 16
7.1 Architecture overview 16
7.2 Installation guide in proprietary environments 16
7.3 Extending the existing pipeline 17
7.4 Configuration and deployment of DIRT 17
7.4.1 Configuration of Database server node 17

7.4.2 Configuration of Web server node ... *17*
7.4.3 Configuration of Grid computing node ... *17*
7.4.4 Deployment of database ... *18*
7.4.5 Deployment on Web server ... *18*
7.4.6 Deployment on grid node ... *18*
7.4.7 Configuration and administration of DIRT ... *19*
7.5 Adding a new pipeline ... 21

8 Limitations ... 22
8.1 File type and size ... 22
8.2 Number of image files per upload ... 22
8.3 Number of images per computation ... 22
8.4 Calibration of one image at a time ... 22

9 Figures ... 23
9.1 Figure S1 ... 23
9.2 Figure S2 ... 24
9.3 Figure S3 ... 25
9.4 Figure S4 ... 26
9.5 Figure S5 ... 27
9.6 Figure S6 ... 28
9.7 Figure S7 ... 29
9.8 Figure S8 ... 30
9.9 Figure S9 ... 30
9.10 Figure S10 ... 31
9.11 Figure S11 ... 32
9.12 Figure S12 ... 32
9.13 Figure S13 ... 33
9.14 Figure S14 ... 33
9.15 Figure S15 ... 34
9.16 Figure S16 ... 35
9.17 Figure S17 ... 36
9.18 Figure S18 ... 37
9.19 Figure S19 ... 38
9.20 Figure S20 ... 39
9.21 Figure S21 ... 40
9.22 Figure S22 ... 41
9.23 Figure S23 ... 42
9.24 Figure S24 ... 43
9.25 Figure S25 ... 44
9.26 Figure S26 ... 45
9.27 Figure S27 ... 46
9.28 Figure S28 ... 46
9.29 Figure S29 ... 47

10 References ... 48

1 DIRT Overview

DIRT is an online web platform to compute root system architecture (RSA) traits from images taken with the DIRT imaging protocol[1]. DIRT facilitates the use of high-throughput computing platform by non-technical plant scientists to compute RSA traits via a set of intuitive web interfaces. DIRT is freely available on the iPlant cyber-infrastructure at http://dirt.iplantcollaborative.org.

2 DIRT pipeline and its parameters

The DIRT RSA trait computation pipeline (Figure S1) is developed in python and is configurable to run on any high-throughput grid computing environment. Its parameters (**Table S1**) are entered via the web interface. The DIRT imaging protocol defines all necessary and optional parameters[1]. Figure S2 shows a sample image taken with the DIRT imaging protocol that contains a circular scale marker with known diameter, an experiment tag with a code, a root crown, and an excised root sample. Note that DIRT supports two different modes for extracting the information on the experiment tag. The first mode allows the extraction of a string via optical character recognition. The second mode allows the use of barcodes or QR codes. Both modes require that the tag is in focus and clearly readable. Failed or incorrect extractions have to be corrected manually.

Table S1: Input parameters to DIRT RSA trait computation pipeline.

Name	Requirement	Description
Masking Threshold	Mandatory	A real value that is used to segment the root structure from the image background.
Scale Marker	Mandatory	The known scale marker diameter allows correction of camera tilting and transforming image coordinates into metric units. For example check the image in Figure S2. A value of 0.0 indicates that no scale marker is present, but the traits will be shown in pixels.
Stem Reconstruction	Optional	Turns on an optional reconstruction of the stem. Maize genotypes occasionally have dark stem parts that have to be reconstructed because they cannot be differentiated from the dark background. We suggest careful use and visual inspection of this feature.
Require Segmentation	Optional	Whether segmentation of foreground and background is required for the image.
Has Root Crown	Optional	Whether the root in the image has crown roots.
Number of Excised Roots	Optional	Number of excised roots present in the image. E.g. the image in Figure S2 has one excised root.

3 DIRT RSA traits

3.1 Common traits

Table S2: Common RSA traits computed by DIRT pipeline. (* annotates traits requested by the user community)

Trait Name	Trait Code	Trait Description
Stem Diameter	DIA_STM	Stem Diameter derived from the medial axis
Simple Stem Diameter*	DIA_STM_SIMPLE	Simple Stem Diameter as calculated in Root Estimator for Shovelomics Traits (REST) from the ETH Zürich[2]
Projected Root Area*	AREA	Number of foreground pixels belonging to the root system. Previously defined in GiA Roots[3]
Average Root Density	AVG_DENSITY	Ratio of foreground to background pixels with in the root shape
Median Tip Diameter	TD_MED	Median Tip Diameter estimated from the medial circle at the tips
Mean Tip Diameter	TD_AVG	Mean Tip Diameter estimated from the medial circle over all detected tips
Median width of root system	WIDTH_MED	Median width of root system measured horizontally from the first to the last foreground pixel
Maximum width of root system	WIDTH_MAX	Maximum with of root system measured horizontally from the first to the last foreground pixel
Accumulated width over 10%-90% percent depth (D-values)	D10-D90	Percentage of width accumulation at 10%-90% depth
Slope of the graph of D-values	DS10 – DS90	Slope at the D10-D90 value that represents the rate of accumulation
Spatial Root Distribution X	RDISTR_X	Spatial distribution of the root shape in x-axis. This is the x component of the vector pointing from the center of the bounding box of the root shape to the center of mass of the root shape
Spatial Root Distribution Y	RDISTR_Y	Spatial distribution of the root shape in y-axis. This is the y component of the vector pointing from the center of the bounding box of the root shape to the center of mass of the root shape
Rooting depth skeleton*	SKL_DEPTH	Rooting depth calculated from the medial axis of the root system. Previously used in GiA Roots [3]
Skeleton Width*	SKL_WIDTH	Width calculated from the medial axis of

		the root system. Previously used in GiA Roots[3]
Number of Root Tip Paths (RTPs)	RTP_COUNT	Corresponds to the overall number of tips detected in the image

3.2 Dicot root traits

Table S3: Dicot RSA traits computed by DIRT pipeline (* annotates traits requested by the user community)

Trait Name	Trait Code	Trait Description
Soil Tissue Angle Range (STA)	STA_RANGE	Range of STA angles present in the root
First Dominant Soil Tissue Angle	STA_DOM_I	Average of the 1st significant peak bin in the histogram of calculated soil tissue angles
Second Dominant Soil Tissue Angle	STA_DOM_II	Average of the 2nd significant peak bin in the histogram of calculated soil tissue angles
Soil Tissue Angle x% 1	STA_25_I, STA_50_I, STA_75_I, STA_90_I	1st dominant angle at 25%, 50%, 75%, 90% of the RTP length
Soil Tissue Angle x% 2	STA_25_II, STA_50_II, STA_75_II, STA_90_II	2nd dominant angle at 25%, 50%, 75%, 90% of the RTP length
Dominant Root Tissue Angle 1	RTA_DOM_I	Average of the 1st significant peak in the histogram of calculated root tissue angles binned in 1 degree steps
Dominant Root Tissue Angle 2	RTA_DOM_II	Average of the 2nd significant peak in the histogram of calculated root tissue angles binned in 1 degree steps
Minimum Soil Tissue Angle	STA_MIN	Minimum Soil Tissue Angle measured over all RTPs
Maximum Soil Tissue Angle	STA_MAX	Maximum Soil Tissue Angle measured over all RTPs
Median Soil Tissue Angle	STA_MED	Median Soil Tissue Angle measured over all RTPs
Root Tissue Angle Range	RTA_RANGE	Range of RTA angles present in the root
Minimum Root Tissue Angle	RTA_MIN	Minimum Root Tissue Angle measured over all RTPs
Maximum Root Tissue Angle	RTA_MAX	Maximum Root Tissue Angle measured over all RTPs
Median Root Tissue Angle	RTA_MED	Median Root Tissue Angle measured over all RTPs
Roots Seg 1*	NR_RTP_SEG_I	Number of RTPs emerging from the Hypocotyl (Root seg 1)

Roots Seg 2*	NR_RTP_SEG_II	Number of RTPs emerging from the taproot (Root seg 2)
Number of adventitious roots*	ADVT_COUNT	Number of adventitious roots estimated as RTP bundles emerging from root seg 1
Number of basal roots*	BASAL_COUNT	Number of basal roots estimated as emerging RTP bundles from root seg 2
Adventitious root angles*	ADVT_ANG	Adventitious root angel estimated from the paths detected in the number of adventitious roots
Basal root angles*	BASAL_ANG	Basal root angles estimated from the paths detected in the number of basal roots
Hypocotyl Diameter*	HYP_DIA	Hypocotyl Diameter estimated over the detected hypocotyl region as the average of diameters of medial circles
Tap root Diameter*	TAP_DIA	Tap root diameter estimated over the detected taproot region as the average of diameters of medial circles
Maximum diameter at 90-100 percent depth	MAX_DIA_90	Maximum diameter found in the interval of 90-100 percent rooting depth
50 percent drop*	DROP_50	Depth value were 50% of the RTPs emerged from the central path (hypocotyl+taproot)
Central path diameter at 25 percent depth	CP_DIA25, CP_DIA50, CP_DIA75, CP_DIA90	Approximation of the tap root diameter at 25%,50%,75%,90% of the rooting depth

3.3 Monocot root traits

Table S4: Monocot RSA traits computed by DIRT pipeline (* annotates traits requested by the user community)

Trait Name	Trait Code	Trait Description
Root Top Angle*	ANG_TOP	Root Top Angle measured between the Random Sample Consensus[4] (RANSAC) fit line at depth of the D10 value and the horizontal soil line.
Root Bottom Angle*	ANG_BTM	Root Bottom Angle measured between the RANSAC fit line at depth of the D80 value and the horizontal soil line.

3.4 Excised root traits

Table S5: Excised root RSA traits computed by DIRT pipeline (* annotates traits requested by the user community)

Trait Name	Trait Code	Trait Description
Average lateral root length	NODAL_LEN	Average length of lateral roots along the central path
Nodal root path length	NODAL_AVG_DIA	Length of the central path along the excised root
Lateral branching frequency	LT_BRA_FRQ	Lateral branching frequency
Mean nodal root diameter	LT_AVG_LEN	Mean nodal root diameter measured along the medial axis of the excised root sample
Lateral mean angle	LT_AVG_ANG	Mean angle of all lateral roots emerging from the excised root sample
Lateral angular range	LT_ANG_RANGE	Range of angles of the lateral root sample
Lateral minimum angle	LT_MIN_ANG	Minimal lateral angle present in all measurements of the excised root sample
Lateral maximum angle	LT_MAX_ANG	Maximal lateral angle present in all measurements of the excised root sample
Distance to first lateral	LT_DIST_FIRST	Distance to first lateral along the medial axis of the excised root
Median diameter of lateral roots	LT_MED_DIA	Median diameter of lateral roots estimated from the medial axis
Mean diameter of lateral roots	LT_AVG_DIA	Mean diameter of lateral roots

4 Output file formats

Computed RSA trait values can be downloaded in two different file formats. Results can be downloaded either as a Microsoft Excel compatible CSV format or the newly developed RSML[5] format for root architecture. Of note, one CSV file is created per computation containing all computed traits of all images, whereas RSML files are created per image. Examples of the two file types containing the computed RSA traits can be downloaded following the links below.

Example CSV file
Example RSML file

5 Public Data

5.1 Image data sets and computations

The iPlant installation of DIRT contains previously published data sets and computations as well as newly contributed data sets. Note, that new traits, features and changes in libraries during the development process might induce smaller numerical inaccuracies in the computations compared to the original versions.

Table S6: Data sets publicly available on DIRT

Name	Previously used in publications	Number of Images	Description
Cowpea Diversity SouthAfrica 2013	[1]	1500	Cowpea Diversity panel collected by James Burridge at URBC, South Africa, 2013
Maize Wisconsin Diversity Panel	[1]	85	Subset of the Wisconsin Diversity Panel collected by Eric Nord, Penn State University and Scott Stelpflug, University of Wisconsin-Madison at URBC, South Africa 2013. (Images taken by Tsi'tso Mokoena).
Technical Error Set Maize	[1]	50	Test set to determine the technical errors in imaging. The maize roots were placed with a supporting structure on the board to minimize placing errors.
Technical Error Set Bean	[1]	50	Test set to determine the technical error in imaging. The bean roots were placed without supporting structures on the board.
Rice grown in gellan gum	[1, 3, 6, 7]	2406	Data produced and used by Iyer-Pascuzzi et. al
Maize Validation Set	Unpublished	99	Maize validation data set collected in Ukulima at the Ukulima Root Biology Center, South Africa 2014.
Barley Diversity Panel	Unpublished	52	Barley Diversity Panel (192 accessions)
German and Australian barley varieties	[8]	64	A comparison study of root system of German and Australian barley varieties. The plants were grown in a semi-hydroponic phenotyping system (Ying L. Chen et al., 2011)
Tomato NILs	Unpublished	568	Solanum lycopersicum x pennellii introgression lines collected summer 2014 from Bradford Research station Columbia, Missouri
Maize Cadriano	Unpublished	20	F4 families of maize

6 Quick start guide

6.1 Access DIRT

The public DIRT installation on the iPlant cyberinfrastructure is accessible at http://dirt.iplantcollaborative.org. The link accesses the DIRT home page (Figure S3).

6.2 Login to DIRT

Click on the 'Login' link on the home page (as show in Figure S3) to log into DIRT. DIRT uses iPlant's Central Authentication Service (CAS) to authenticate its users. Therefore, it's available to all iPlant's registered users. By clicking on the 'Log in' button (Figure S4), will direct the user to the iPlant authentication services interface. Alternatively, using the 'Cancel iPlant Login' link (as shown in Figure S4) will direct the user to the DIRT authentication services interface. The iPlant authentication interface also provides link to sign up for an iPlant account.

6.3 Root image collection

A root image collection (aka **collection**) is a container that holds a set of root images belonging to an experiment and its metadata. Each root image in the collection can also be tagged with its own metadata. All functionalities (mentioned in following subsections i.e. 6.3.1, 6.3.2, etc.) associated to root image collection(s) are available to a logged in user via '**ROOTS**' menu and its sub-menu. An anonymous user can only view publicly available collections, its metadata and associated root images.

6.3.1 Create root image collection

A user first creates a root image collection, in order to store and manage root images. To create a root image collection, select '**CREATE ROOT COLLECTION**' sub-menu as shown in Figure S5 and fill in values for the mandatory metadata fields. On successful submission of this form, the user will be navigated to the image upload screen as described in the next section.

6.3.2 Upload images to a collection

On creation of a new root image collection the user is navigated to the image upload interface shown in Figure S6. A maximum of 200 image files of type png, gif, jpg and jpeg can be uploaded at once. This restriction is in account of the limitations of browser's support for the http POST data transfer protocol. Steps to upload images are:

1. Click on 'Add files' link on this interface to add images from your local computer
2. Click on 'Start upload'
3. Enter values for the mandatory fields (i.e. with *) and optional fields as applicable
4. Scroll down and click on 'Add to collection' button (not seen in Figure S6).

While images are being added to the collection by a background process, the user is navigated to the root image collection's view interface with a message i.e. '*# uploaded images are being added to this collection*'. The collection's view interface can be refreshed manually to see the recently added images or can be visited at a later time.

6.3.3 View root image collections

All collections managed by a user are accessible by visiting the '**ROOTS**' menu and then clicking on the '**My**' tab (Figure S7).

6.3.4 Share root image collection

Root image collections managed by a user can be shared publicly or privately with collaborators.

6.3.4.1 Share a collection with public

Root image collections are by default private i.e. accessible only by the owner or manager. To mark a root image collection public:

1. Click on the '**ROOTS**' menu item
2. Select the collection from the '**My**' tab (Figure S7)
3. Click on the 'Edit' tab (Figure S8)
4. Scroll down and expand the 'Collections settings' section
5. Choose public under collection visibility section and click save

Public collections are visible to all the visitors of the site including non-registered or anonymous users.

6.3.4.2 Share the collection with collaborators

To share a root image collection privately with collaborators (i.e. other registered users of DIRT)

1. Select a collection from the '**My**' tab by visiting the '**ROOTS**' menu
2. From root image collections view page click on the 'Group' tab (Figure S9)
3. Click on the 'Add people' link.

The 'Add people' link will bring up an interface to search for a user and add the user to the collection. To manage membership of the existing users, click on the 'People' link as shown in Figure S9. User name of all registered users can be accessed by visiting the 'ABOUT' menu.

6.3.5 Download images from a collection

A registered user can download root images from a public collection or from a collection he/she is a member of. To download images:

1. Select a collection from the '**Public**'/'**My**' tab by visiting the '**ROOTS**' menu
2. Selecting a collection will bring up the collection view page (Figure S10)
3. Select images of choice and click on the '**Download Images**' button (Figure S10)
4. Selected images will be downloaded as a 'ZIP' file. Based on the user's browser settings it will either open up an window asking user to save the zip file or it will save the zip file to the download directory.

6.3.6 Download image metadata of a collection

Metadata associated with images of a collection can be downloaded from the collection view interface (Figure S10). The metadata of the selected images will be downloaded in CSV file format. The following lines shows contents of a sample metadata CSV file that can be edited via an ASCII text editor such as Notepad or Microsoft Excel.

Image ID, Image Name, Genus, Species, Family, Dry Biomass, Fresh Biomass, SPAD, Age, Resolution(pixels/mm)
17951,DSC_0042.JPG,Vigna,unguiculata,Fabaceae,0,0,0,0,0
17952,DSC_0043.JPG,Vigna,unguiculata,Fabaceae,0,0,0,0,0

First line of the CSV file represents the field labels and subsequent lines represent the metadata values of the images. This file can also be used to '**Add metadata**' to the images of a collection. To download metadata of images of a collection:

1. Select a collection from the '**Public**'/'**My**' tab by visiting the '**ROOTS**' menu
2. Selecting a collection will bring up the collection view page (Figure S10)
3. Select images of choice and click on the '**Download Metadata**' button (Figure S10)

6.3.7 Add metadata of images in a collection

Each image in DIRT has its own metadata. Image metadata can be added either by editing each image or in batch by uploading a CSV file per collection containing metadata information about each image. To add/update/edit metadata of each image:

1. Go the collection view page as shown in Figure S10
2. Select an image by clicking on the link shown below the image
3. On image view page click on the 'Edit' tab to add/update its metadata

Metadata batch upload for multiple images of a collection:

1. Download metadata file of the selected images or all images in a collection (see section **6.3.6**)
2. Edit and update the downloaded CSV file with appropriate metadata values. Optionally, you can add additional key names at the end of the header and fill in the corresponding values.
3. Click on the 'Add Metadata' on the collection view interface (Figure S10)
4. It will bring up the metadata upload interface as shown in Figure S11
5. Enter a title, select the updated metadata CSV file by clicking on the 'Browse' button and then click on 'Upload' and finally click 'Save'.

6.4 Marked image collection

Marked image collection (aka **Marked Collection**) is a functionality that allows users to create virtual image collection by mixing images from different physical root image collections. This is also a required functionality for the calibration (defined in **Section 6.5** of the Supplementary Information) and RSA trait computation (defined in **Section 6.6** of the Supplementary Information). The marked collection functionality is only privately available to a registered user. A user can access all of his/her marked collections by visiting the '**MARKED COLLECTIONS**' menu as shown in Figure S14. Marked collection functionality is also available on the collections view page as shown in Figure S10, where a user can add selected images to a new or existing marked collection.

6.4.1 Create a marked collection

To create a new marked collection:

1. Go to the collection view page by selecting a collection from your list (Figure S7)
2. Select (all or some) images from the collection view page (Figure S10)
3. Click on 'Add to Marked Collection' button
4. The click will open the marked collection interface as shown in Figure S12, select option 'No' and enter a name for the new marked collection and click on the 'Next' button.

6.4.2 Add images to an existing marked collection

To add images to an existing marked collection, follow the same procedure as mentioned in Section **6.4.1** of the Supplementary Information, in step 4 instead on selecting option 'No', select 'Yes' as shown in Figure S13 and then choose a marked collection from the select list. If the select list has zero items, change your option to 'No' to create a new marked collection.

6.4.3 View marked collections

To view the list of available marked collections, click on the 'MARKED COLLECTIONS' menu item as show in Figure S14. The user can delete an existing marked collection by selecting a collection from the list and clicking on the 'Delete Marked Collections' button.

6.5 Image Threshold Calibration

The RSA trait computation pipeline available on DIRT, requires the user to provide segmentation threshold value as a parameter. A user can determine the threshold value by using image threshold calibration (aka **Calibration**) functionality. Calibration enables DIRT users to visually inspect calculated masks of a selected root image for a set of threshold values of 1, 3, 5, 10, 15 and 20. Hence, users without technical background can choose appropriate threshold values for the computation. A user can access this functionality by clicking on the '**CALIBRATIONS**' menu and its sub-menu '**CALIBRATE**'. DIRT enables calibration of one image at a time. If a user wants to calibrate the thresholds for multiple images, he/she has to calibrate several images individually and compare their results visually. The calibration functionality can also be accessed from the 'Create Computation' interface (defined in Section **6.6.1** of the Supplementary Information).

6.5.1 Calibrate threshold value of an image

To calibrate an image, click on the 'CALIBRATE' sub-menu as shown in Figure S15.
It works in two steps:

1. Select a marked collection whose image needs calibration and click 'Go' as shown in Figure S15.
2. Choose an image and click on 'Calibrate Threshold'. Depending on the image resolution this process may take several minutes (may be more than 15 minutes). During this computation the user will see the interface as shown in Figure S16. Do not close the browser window while it computes. Moving the focus away from the browser, won't show the message as seen in Figure S16, but the computation is still running for the selected image and the page will refresh with masked images after its completion. Closing the browser window will terminate the computation.

On completion of the calibration, the user will be presented with binary masked images and their corresponding threshold values (Figure S17). The user can select the appropriate masked image and navigated to the 'Create computation' page with corresponding threshold value.

6.5.2 View calibrations

A user can access all past calibrations (Figure S18) by clicking on the '**CALIBRATIONS**' menu. The calibration list interface also allows the user to delete a past calibration. To delete a calibration, select the appropriate row and click on the 'Delete Calibrated Images' button.

6.6 RSA Trait Computation

RSA trait computation (aka **Computation**) enables the user to compute the whole or sub-set of traits defined in Section 3 of the Supplementary Information, for all the images in a marked collection. A user can access this functionality by clicking on the '**COMPUTATIONS**' menu and its '**CREATE COMPUTATION**' sub-menu.

6.6.1 Create a computation

The create computation interface enables the user to transfer the images of a marked collection to the high-throughput computing system and run the DIRT RSA trait computation pipeline on the images of the marked collection. DIRT on the iPlant cyber infrastructure submits images to TACC's stampede environment (https://www.tacc.utexas.edu/systems/stampede) to compute multiple images at once. In order to initiate the transfer, a user has to navigate to the create computation interface. This interface is accessible as the 'CREATE COMPUTATION' item in the 'COMPUTATIONS' menu (see Figure S19). For detailed description about the pipeline

parameters, we refer to Section S2 of the Supplementary Information. For an appropriate masking threshold value the user can access the calibration functionality, from this interface by clicking on the 'Calibrate' link as shown in Figure S19. By default all RSA traits are marked for computation. The user can change this setting by expanding the appropriate sections (i.e. Common Traits, Dicot Root Traits, etc. as shown in Figure S19) and then checking and unchecking the required traits. On successful submission the user will be navigated to computations list page with a message i.e. '*Submitting jobs to the grid! Computation <name> has been created*'. Note, that also images can be computed with no scale marker (e.g. Barley Diversity panel). However, the output will be in pixels.

6.6.2 View computations

A user can view his/her computations by clicking on the 'COMPUTATIONS' menu and then selecting 'My' tab interface as shown in Figure S20.

6.6.3 Share a computation

Similar to a collection, a computation can be shared publicly with all or privately with trusted collaborators.

6.6.3.1 Share with public

Similar to root image collections, computations are marked private by default. The owner of the computation can declare it public by editing the computation as shown in Figure S21 and changing its 'Computation Settings' to public. To mark a computation public:

1. Click on the 'COMPUTATIONS' menu item
2. Select the computation from the 'My' tab (Figure S20)
3. Click on the 'Edit' tab (Figure S21)
4. Scroll down and expand the 'Computation settings' section
5. Choose public under collection visibility section and click save

Public computations are visible to all the visitors of the site including non-registered or anonymous users.

6.6.3.2 Share privately with collaborators

To share a computation privately with collaborators (i.e. other registered users of DIRT):

1. Select a computation from the 'My' tab by visiting the 'COMPUTATIONS' menu (Figure S20)
2. From computations view page (Figure S23) click on the 'Group' tab
3. Click on the 'Add people' link.

Add people link will bring up an interface to search for a user and add the user to the computation. To manage membership of the existing users, click on the 'People' link.

6.6.4 Cancel a computation

A user can cancel a submitted or running computation anytime during its execution. To cancel a running computation,

1. Go to the computations list page by clicking on the 'COMPUTATIONS' menu (Figure S20)
2. Select a computation whose status is either 'Submitted' or 'Running'
3. On the computations view page as shown in Figure S22 click on the 'Cancel' button.

6.6.5 Download computed RSA traits

6.6.5.1 Download computed traits of whole computation

After a computation has completed successfully, the computed traits are available for download in the form of a CSV file. To check the contents of a sample CSV file refer we refer to Section S6. The CSV file and image metadata file are available for download on the computation's view page as shown in Figure S23.

6.6.5.2 Download computed traits for one image of a computation

Besides a CSV file containing the computed traits for the whole computation, each image is additionally associated with a RSML[5] file. To check a sample RSML file, we refer to Section S6. To access the RSML file:

1. Select one by clicking on the link present under the masked image on the computation view interface (Figure S23)
2. It will navigate to the individual image's computation view page containing link to the RSML file (Figure S24)

6.7 User quota

DIRT allocates a configurable storage and file transfer quota to its users. Each DIRT user on iPlant, has a total storage limit of 100 GB and daily computation (i.e. file transfer/image processed) limit of 10 GB. The user can access this information by visiting the user profile page (as shown in Figure S25) by clicking on the user name at top right corner. If more iPlant resources become available to DIRT, the limit will be increased.

7 Developer guide

7.1 Architecture overview

DIRT is a multi-tiered integrated online platform developed using the Drupal framework[9]. DIRT a three tier platform; (1) **client** tier represent the web browser user interface,(2) **processing** tier represents the Drupal[9] modules and the image processing pipeline, and (3) **storage** tier represents the database and file systems.

7.2 Installation guide in proprietary environments

DIRT is an open source platform developed on the open source content management system Drupal. Hence, DIRT can be installed on any proprietary environment and be extended to meet specific user requirements. For system requirements and installation instructions, we refer to Drupal's online documentation available at https://www.drupal.org/documentation/install.

In order to install DIRT on your local environment, two prerequisites have to be installed and configured as described below:

1. *Web server:* To configure the web server follow the Drupal installation document mentioned in the previous paragraph. Once you have a Drupal instance running, download the DIRT source from http://dirt.iplantcollaborative.org/sites/default/dirt_files/dirt-src.tar and extract it to your system. This archive file contains the 'dirt.sql' file and 'public_html' directory. Load the 'dirt.sql' to your local database instance and copy the contents of the public_html to your local web root directory. Make sure that the 'settings.php' file is updated with correct database credentials to connect to your local database.
2. *DIRT pipeline:* Download DIRT pipeline from https://github.com/abucksch/DIRT and install and configure required software stack on both the web server and the grid computing platform.
3. *Install and configure interface scripts:* Log in to your local DIRT site as the administrator and go to 'DIRT Server Configuration' interface under 'Configuration' menu as shown in Figure S26. Enter your grid server and web server details and save. Similarly access 'DIRT Quota Configuration' interface (Figure S27) and enter the daily quota limit and save. Log in to your grid computing environment and download and extract this file http://dirt.iplantcollaborative.org/sites/default/dirt_files/dirt-server-scripts.tar. On extraction this file will create a directory called 'src'. Make sure that 'src' directory is directly under the server home directory as configured using the 'DIRT Server Configuration' page. The 'src' directory contains two sub-directories called 'python' and 'scripts'. Update 'dirt_job.slurm' and 'submit_dirt_jobs.sh' scripts in 'scripts' directory as per your local grid computing environment and scheduler. Log in to your web server system, go to the web server's script directory as configured on the 'DIRT Server Configuration' page and download and extract this http://dirt.iplantcollaborative.org/sites/default/dirt_files/dirt-web-scripts.tar file.

7.3 Extending the existing pipeline

Existing DIRT image processing pipeline can be extended to compute new RSA traits. To extend the pipeline take following steps:

1. Download the python source code from https://github.com/abucksch/DIRT and make necessary changes to your need.
2. Log in to Drupal as an administrator and update the existing content called 'DIRT_Py' to reflect the new traits.
3. Log in to Drupal as an administrator and add new fields corresponding to the new traits to Drupal content types 'Computation' and 'DIRT Output'.
4. Update the DIRT Drupal modules 'dirt_run_computation' and 'dirt_job_status' to handle new traits.

7.4 Configuration and deployment of DIRT

7.4.1 Configuration of Database server node

The MySQL database has to be installed and a database must be created for DIRT. We recommend that one follow the MySQL and Drupal installation instructions/guide to configure database.

7.4.2 Configuration of Web server node

The node hosting web server is configured with PHP, Apache and Python to run Drupal and trait computation pipeline. Besides all PHP libraries (i.e. bz2, gmp, zlib, gd, libjpeg, libtiff, libpng3, libxml, t1lib5, libmcrypt, libmhash, wget, bzip2, ming, pdflib, php-bcmath, date, dba, dom, json, ssh, scp, mysql etc.). File_Archive libraries are also mandatory to be installed. After the PHP installation, the following configuration are suggested to be updated in php.ini file:

- max_execution_time=120
- memory_limit=512M
- post_max_size=1024M
- upload_max_filesize = 10242M
- max_file_uploads = 20

After installation of Python and dependent modules (see source code for dependencies) read access to all libraries has to be given on the file system level to all the system users.

The directory structure, as shown in Figure S28, is created to organize and store the contents of DIRT modules. Write permission must be granted on the 'temp' directory. The 'logs' directory will be used to enable web server access and store error log files. The 'public_html' directory will be used to administrate all web server contents including all Drupal contents. The 'calibrate' directory under 'scripts' contains the trait computation scripts. The 'job_status' directory contains the shell scripts to process trait computation outputs. The Apache web server is configured with a virtual host to serve content from the 'public_html' directory and write log to the 'logs' directory.

7.4.3 Configuration of Grid computing node

DIRT computes RSA traits in high-throughput fashion. TACC's STAMPEDE environment was configured with the required Python libraries to run the DIRT pipeline (see DIRT source code for dependencies). The directory structure as shown in Figure S29 must be created on the grid node to house python and shell scripts. The 'data' and 'out' directory under $WORK (working

directory) provides storage for the original image data and output data from the pipeline during and after trait computation.

7.4.4 Deployment of database

For the iPlant DIRT installation, the database is seeded and populated during configuration and module installation. The same procedure can be used by folks wishing to install, setup and instance DIRT on their local/private proprietary environment. To deploy the database; (a) download the DIRT source archive from the DIRT website at http://dirt.iplantcollaborative.org/sites/default/dirt_files/dirt-src.tar to the database server node, (b) extract the archive and (c) load the dirt.sql file to the database created earlier.

7.4.5 Deployment on Web server

For the iPlant DIRT installation, the web server components were developed de novo as part of configuration process. In order to install, setup and instance DIRT on a local/private proprietary environment: (a) download the DIRT source archive from the DIRT website at http://dirt.iplantcollaborative.org/sites/default/dirt_files/dirt-src.tar to the web server node, (b) extract the archive to the 'public_html' directory created earlier and (c) edit the 'settings.php' file under 'public_html/sites/default' directory and update the database configuration setting parameter appropriate to your installation.

Download and extract the web server scripts from the site at http://dirt.iplantcollaborative.org/sites/default/dirt_files/dirt-web-scripts.tar to the 'scripts' directory created earlier. Make sure that these scripts have execution rights and 'calibrate' directory contains python scripts and 'job_status' directory contains following shell scripts:

- dirt_clean.sh
- dirt_extract.sh
- dirt_read_log.sh

DIRT web server schedule the 'dirt_job_status' rules modules via cron to run in every 10 minutes to check the status of the active computations on the grid. The web server administrator should setup a cron job on the web server to trigger every 10 minutes with the following command:

10 * * * * /usr/bin/wget -O - -q -t 1 http://<DomainURL>/cron.php?cron_key=<CronKey>

Make sure to change the *DomainURL* and *CronKey*.

7.4.6 Deployment on grid node

Extract the archive file http://dirt.iplantcollaborative.org/sites/default/dirt_files/dirt-server-scripts.tar to the grid node's home directory. It will create and copy the files to appropriate directories (see configuration section). Make sure that the 'src/python/1.1' directory contains the python scripts and 'src/scripts' directory contains following shell scripts:

- cancel_jobs.sh
- clean_output.sh
- prepare_data.sh
- check_job_status.sh
- dirt_job.slurm
- prepare_output.sh
- clean_all.sh
- submit_dirt_jobs.sh

Modify these scripts to meet your grid job scheduler's commands.

7.4.7 Configuration and administration of DIRT

After the successful configuration and deployment the modules on respective nodes, start your database and Apache server. Access your instance's URL (i.e. Apache virtual hosts name) in a browser and login with your administrator credentials. If you have setup the instance from the existing DIRT source, all the required modules, contents, rules, workflows, themes, views, etc. should have already been enabled for you. Although everything is preconfigured, we briefly describe in this section all custom DIRT modules and some important community contributed modules whose source code was updated to meet DIRT specifications. We recommend administrators to familiarize themselves with all the enabled Drupal modules in DIRT.

7.4.7.1 DIRT theme

The DIRT platform has adopted and used a Drupal community contributed module called 'Danland' to theme its user interfaces. Detail information about this module can be found on the Drupal website at https://www.drupal.org/project/danland. We have tweaked its source code and customized it to provide the current user interfaces. This module can be administered from the DIRT's 'Appearance' administration menu. This module resides on the web server node under 'public_html/sites/all/themes/danland/' directory. Its CSS and page template files have been modified to achieve the current look and feel. We have also added a custom a JavaScript file called 'jquery.dirt.js' to alter validation on computation form. Therefore, this module should not be auto updated to new version by the webmaster or the administrator.

7.4.7.2 DIRT content model

After the basic core setup, the content model for the DIRT platform must be defined and configured to handle system requirement specifications. In Drupal terms, the 'Content Types' (analogous to a class in object oriented concept) must be defined. The DIRT content model depends on many Drupal modules (like *Entity, Entity Reference, Organic Groups, etc.*). These modules must be deployed and configured before defining new custom content types. All these modules reside on the web server node at 'public_html/sites/all/modules' directory and they can be accessed via administrative 'Modules' menu item. All custom DIRT content types can be accessed and managed via administrative 'Structure > Content Types' menu item.

7.4.7.3 Batch upload of root images to a collection

To enable batch upload of images to a collection DIRT has used and adopted a community contributed module called 'bulk_media_upload'. Detail information about this module can be found on the Drupal's website. This module resides on the web server node under 'public_html/sites/all/modules/bulk_media_upload' directory. The source code of this module has been modified to meet DIRT specifications. This module should not be auto upgraded. This module can be configured by the administrator by visiting the 'Media/Bulk Media Upload Settings' page under 'Configurations' menu.

7.4.7.4 DIRT grid server configuration

This is a custom DIRT administration module that resides on the web server node at 'public_html/sites/all/modules/dirt_server_conf' directory. This enables to store grid computing server details and web server location details to be used by other modules like grid job submission module across the system. Other modules of the platform depend on this administration module. Hence dependent modules cannot be deployed and/or enabled without the administration module. The administrator should first configure and save server configuration details by accessing the 'Configuration > DIRT Server Configuration' menu item.

7.4.7.5 DIRT user quota configuration

Similar to the previous module, it is also a custom DIRT administration module that resides on the web server node at 'public_html/sites/all/modules/dirt_transfer_quota' directory. As the name suggests, it allows storing and parameterizing the daily file transfer quota for a user from web server node to the grid computing node. It can be accessed by the administrator via 'Configuration > DIRT Quota Configuration' menu item. Besides daily transfer quota DIRT also has option to configure total file storage quota for a user. This quota can be set per each user role by visiting the 'People > Disk Quota' administration menu item.

7.4.7.6 DIRT grid job submission

This is a custom DIRT rules module that depends on 'dirt_server_conf' and 'rules' module to handle RSA trait estimation and computation on the configured grid node as an asynchronous process. This module is available on the web server node at 'public_html/sites/all/modules/dirt_run_computation' directory. This module is used to define a DIRT workflow rule, that is triggered on creation or save of a new content of type 'Computation'. On start of the workflow rule, this module starts a background process, that reads details from the computation object, prepares and archive of the image data along with pipeline parameters and selected traits, estimates the average grid computation wall time and processor requirements based on the image size and count, receives a secure connection with the grid node, transfers the content to grid node, executes scripts on grid node to extract and prepare the data for computation and executes scheduler job submission script, wait for it to be submitted, gets the grid job identifier, updates the database and notifies the user. These workflow rules can be accessed and configured by visiting the 'Configuration > Workflow > Rules' administrative menu item.

7.4.7.7 DIRT grid job status check

Similar to the previous module, it is also a custom DIRT rules module that depends on 'dirt_server_conf' and 'rules' module. This module is responsible for tracking a grid job and transferring the generated output from the grid node to the web server node, updating the database and notifying the user on job status. This workflow rule is also triggered on creation of a new content of type 'Computation'. Unlike the previous module, it is configured and scheduled to run every 10 minutes, until the grid job completion. This module uses the scripts on web server nodes and grid server nodes to perform its tasks. This module resides on the web server node at 'public_html/sites/all/modules/dirt_job_status' directory. Similar to the previous module it can be accessed and configured via 'Rules' administrative menu item.

7.4.7.8 DIRT calibrate threshold

This is a custom DIRT module that enables image threshold calibration. It depends on 'dirt_server_conf' and 'views_bulk_operation' (a community contributed module). It resides on the web server node at 'public_html/sites/all/modules/dirt_threshold_vbo' directory and uses RSA trait estimation pipeline components on the web server node.

7.4.7.9 DIRT marked collection

This module is responsible for creating/adding images to a 'Marked Collection' from a 'Collection'. It resides on the web server node at 'public_html/sites/all/modules/dirt_vc_vbo' directory and depends on 'views_bulk_operation' module.

7.4.7.10 DIRT metadata upload and download

Metadata upload to a 'Collection' and download from a 'Collection' is handled by two different modules called 'dirt_process_metadata' and 'dirt_metadatadl_vbo' respectively. Both these modules reside on the web server node. Metadata upload module is a rules module that resides at 'public_html/sites/all/modules/dirt_process_metadata' directory and depends on 'rules' and 'background_process' community contributed module. This module is triggered on upload of a metadata file to a collection. The metadata download module resides at 'public_html/sites/all/modules/dirt_metadatadl_vbo' directory and depends on 'views_bulk_operation' module.

7.4.7.11 DIRT grid job cancellation

This module is responsible for cancelling a submitted or a running job on the grid environment. It resides on the web server node at 'public_html/sites/all/modules/dirt_cancel_job' directory and depends on 'dirt_server_conf' module.

7.4.7.12 DIRT bulk image download

Like the metadata download module, this is a custom DIRT module responsible for downloading images as an archive (zip) file from the 'Collection'. It depends on 'views_bulk_operation' module and resides at 'public_html/sites/all/modules/dirt_download_image_vbo' directory on the web server node.

7.5 Adding a new pipeline

The DIRT platform design allows to add new RSA trait computation pipeline for existing traits or new traits. In addition to tasks performed in the user interface, adding a new pipeline requires some updates (including source code update) to the following nodes of DIRT platform:

1. Grid node
    - The new pipeline must be manually installed and configured on the grid computing environment.
    - New scheduler specific job submission scripts must be created to handle its parameters.
2. Web server node
    - A new content of type 'Image Processing Pipeline' must be added via the administrative 'Content > Add content > Image Processing Pipeline' menu item.
    - If the new pipeline introduces traits, these traits must be added to the 'Computation' and 'DIRT Output' content types.
    - If the pipeline requires any specific parameters, those fields must be added to the 'Computation' content type.
    - User interface validation scripts must be added to 'Computation' forms to show parameter fields with respect to a pipeline.
    - The 'dirt_run_computation' and 'dirt_job_status' modules must be updated to handle new pipeline, traits and pipeline parameter fields.

If the pipeline generates output in any proprietary format, 'dirt_job_status' module needs to be updated accordingly to process the pipeline output file types.

8 Limitations

Even though DIRT is a high-throughput online scalable platform, it has technological constraints. The iPlant installation and the easy web access to high-throughput computing system restrict data upload and computation time. Furthermore the current algorithm restricts RSA trait computation to images taken according to DIRT imaging protocol [1]. In the following subsections we describe the limitations of the publicly available iPlant installation of DIRT. DIRT on iPlant installation can be accessed via any web browser; however it has been tested on Google Chrome (Version 43), Firefox (Version 38), Internet Explorer (Version 11) and Safari (Version 8). The web browser's privacy has to be set to accept third-party cookies and allow the site to run JavaScript.

8.1 File type and size

The iPlant installation of DIRT exclusively accepts image files of types png, gif, jpg and jpeg. While no restrictions are imposed on the image resolution, single image file is not allowed to exceed 1 GB.

8.2 Number of image files per upload

The batch upload to a root image collection is limited to 200 images at a time. As explained earlier in Section 6.3.2 of the Supplementary Information, this restriction gives end-users easy and quick web experience, while taking into account the limitations of http POST protocol, browser support and internet speed.

8.3 Number of images per computation

The RSA trait computation pipeline runs on STAMPEDE [10] environment at TACC. Stampede limits wall-clock time of productions queue’s to 48 hrs and the processor number to 4000. The priority of allocated resource for a computation is decided by the underlying **S**imple **L**inux **U**tility for **R**esource **M**anagement (SLURM) batch environment. Therefore, jobs requesting more resources may have to wait longer for resource allocation. DIRT estimates appropriate wall-clock time and processor needs for each submitted computation based on the image size and number. Currently, DIRT does not have a limit on the number of images that can be submitted per computation, but we recommend our users not to submit a computation of marked collection with more than 4000 images.

8.4 Calibration of one image at a time

The calibration functionality of DIRT is a synchronous process that computes mask images for six different threshold values per image simultaneously and uses web server memory. Depending upon the image size this process takes from seconds to couple of minutes to finish. Therefore, as of this release DIRT only allows the calibration of one image at a time. Users wanting to calibrate multiple images of a marked collection have to bear with this limitation. However, it is possible to calibrate multiple images (one at a time) and visually explore the threshold values.

9 Figures

9.1 Figure S1

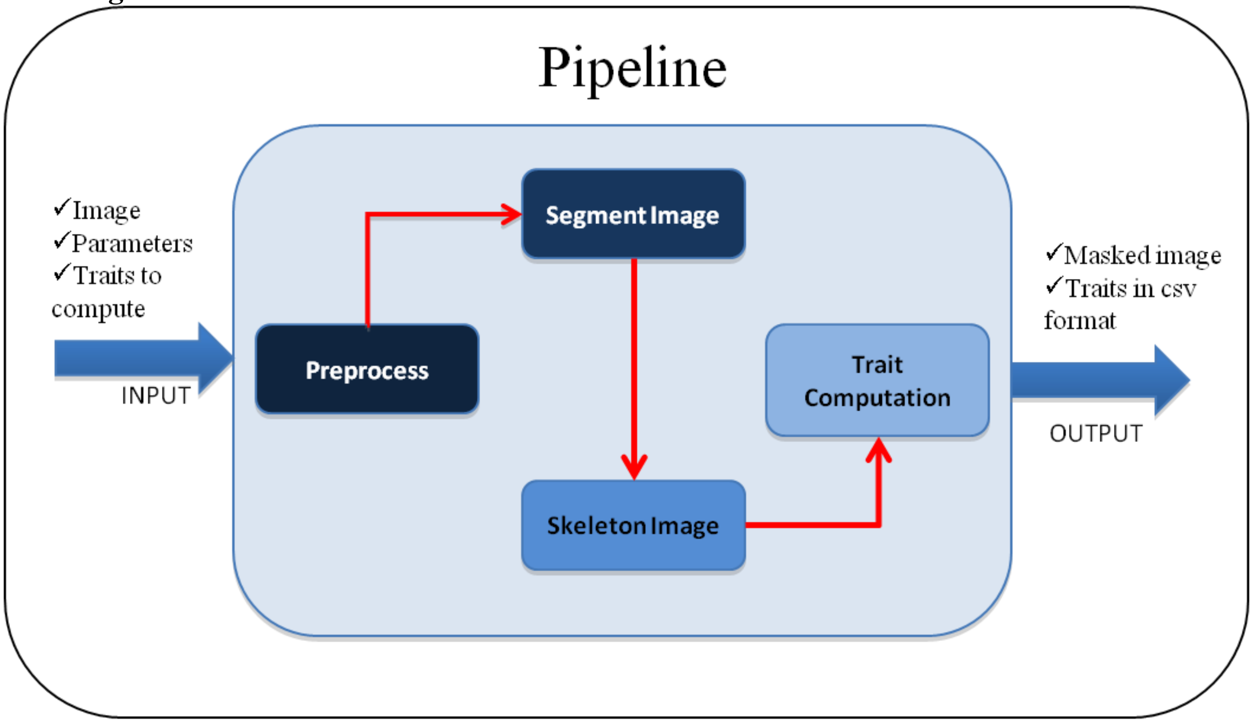

Figure S1: A schematic showing different steps of the DIRT RSA trait computation pipeline

9.2 Figure S2

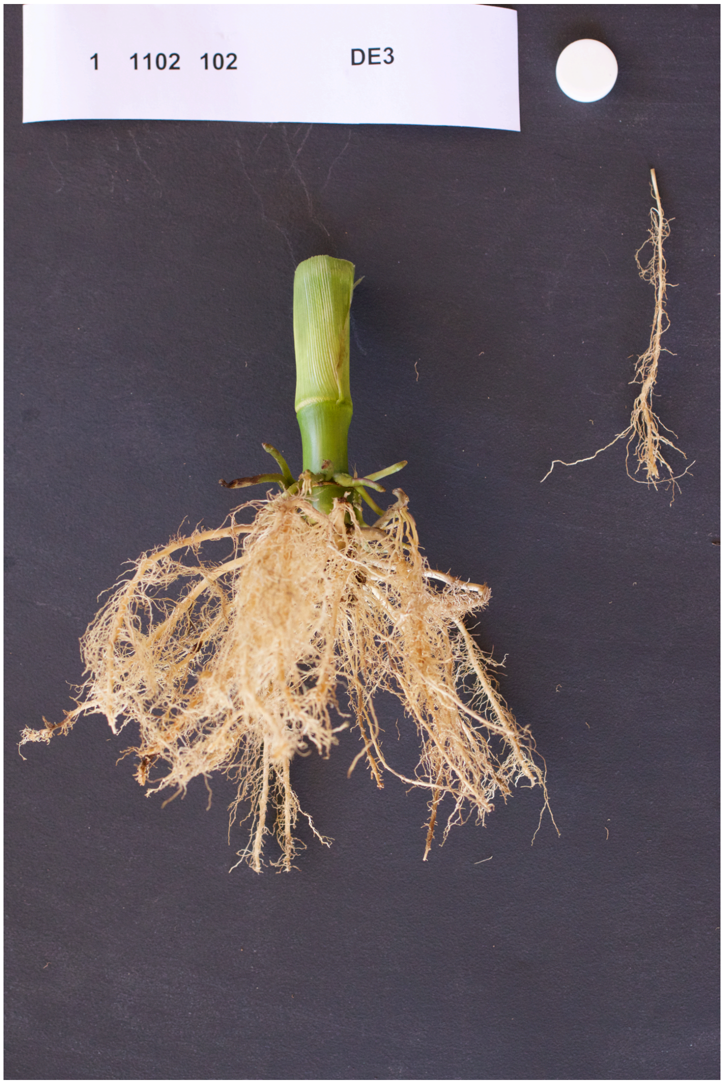

Figure S2: An example image that uses all DIRT image acquisition features[1]. The round scale marker is needed to recalculate pixels into units. The rectangular experiment tag is automatically detected and its content (letters or barcode) is stored in the result file. The root crown and the excised roots are separately analyzed.

9.3 Figure S3

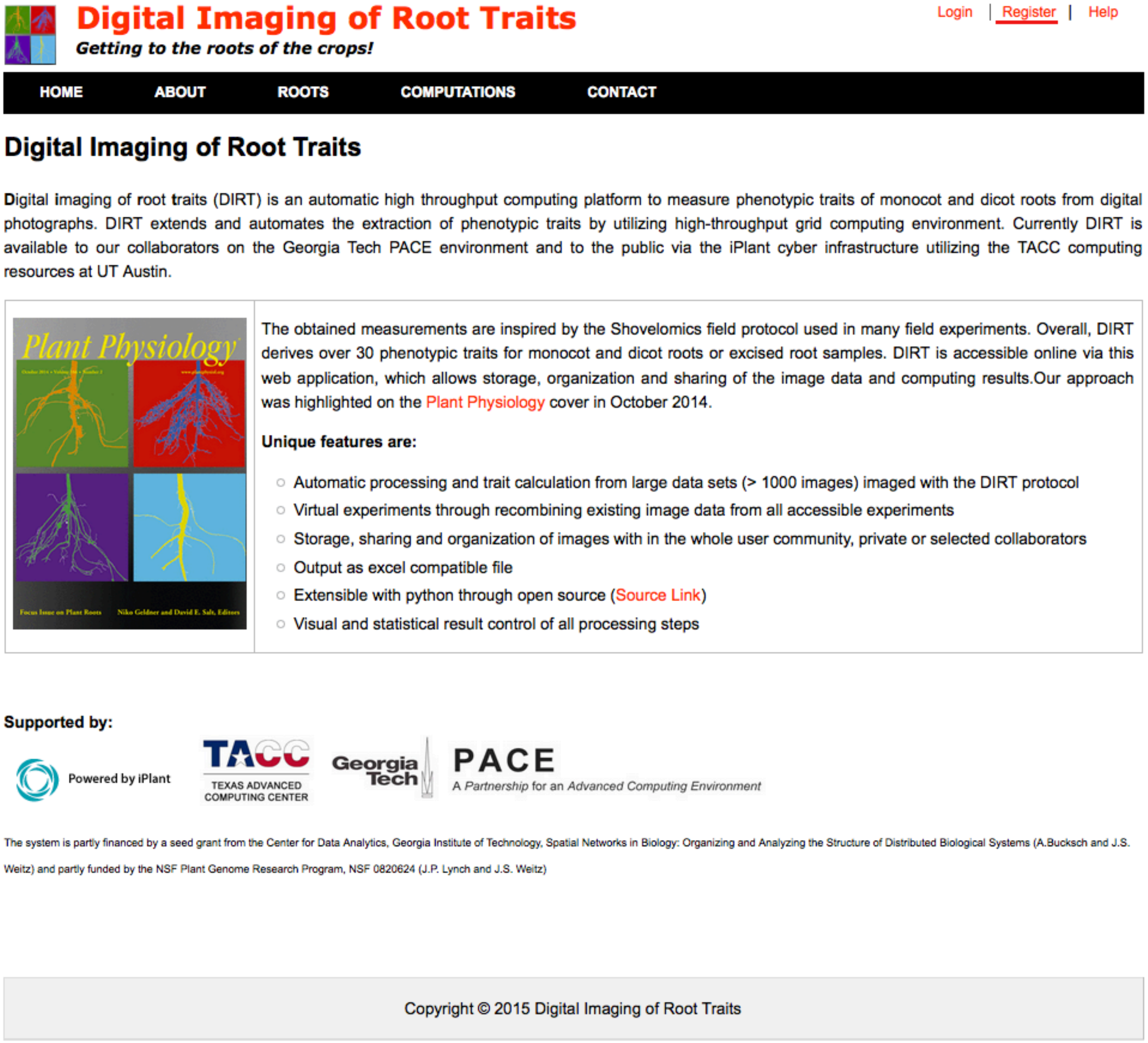

Figure S3: Screenshot of the DIRT home page.

9.4 Figure S4

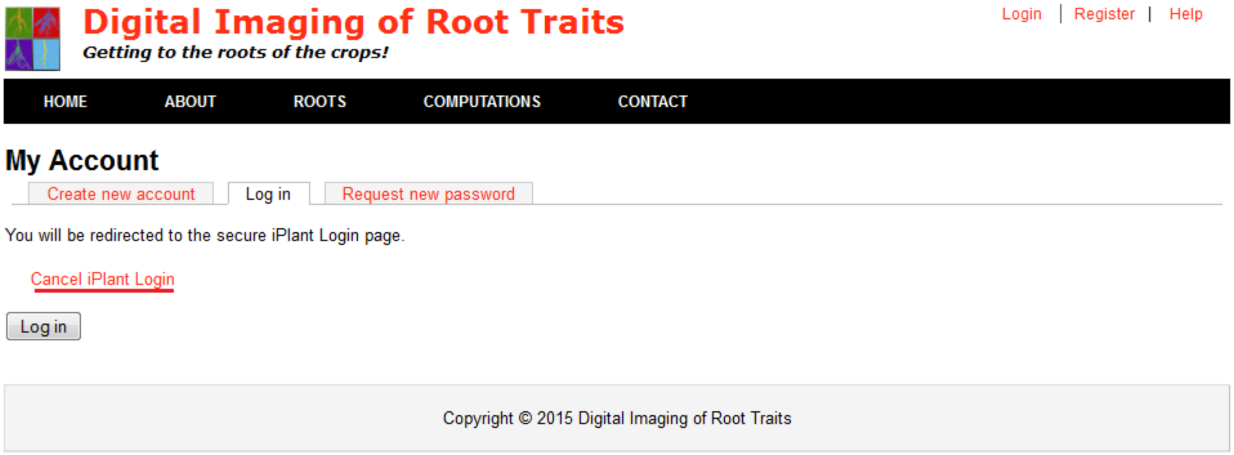

Figure S4: Screenshot of the DIRT user's login page. This is shown after clicking on the 'Login' link available at the top right corner of the DIRT home page.

9.5 Figure S5

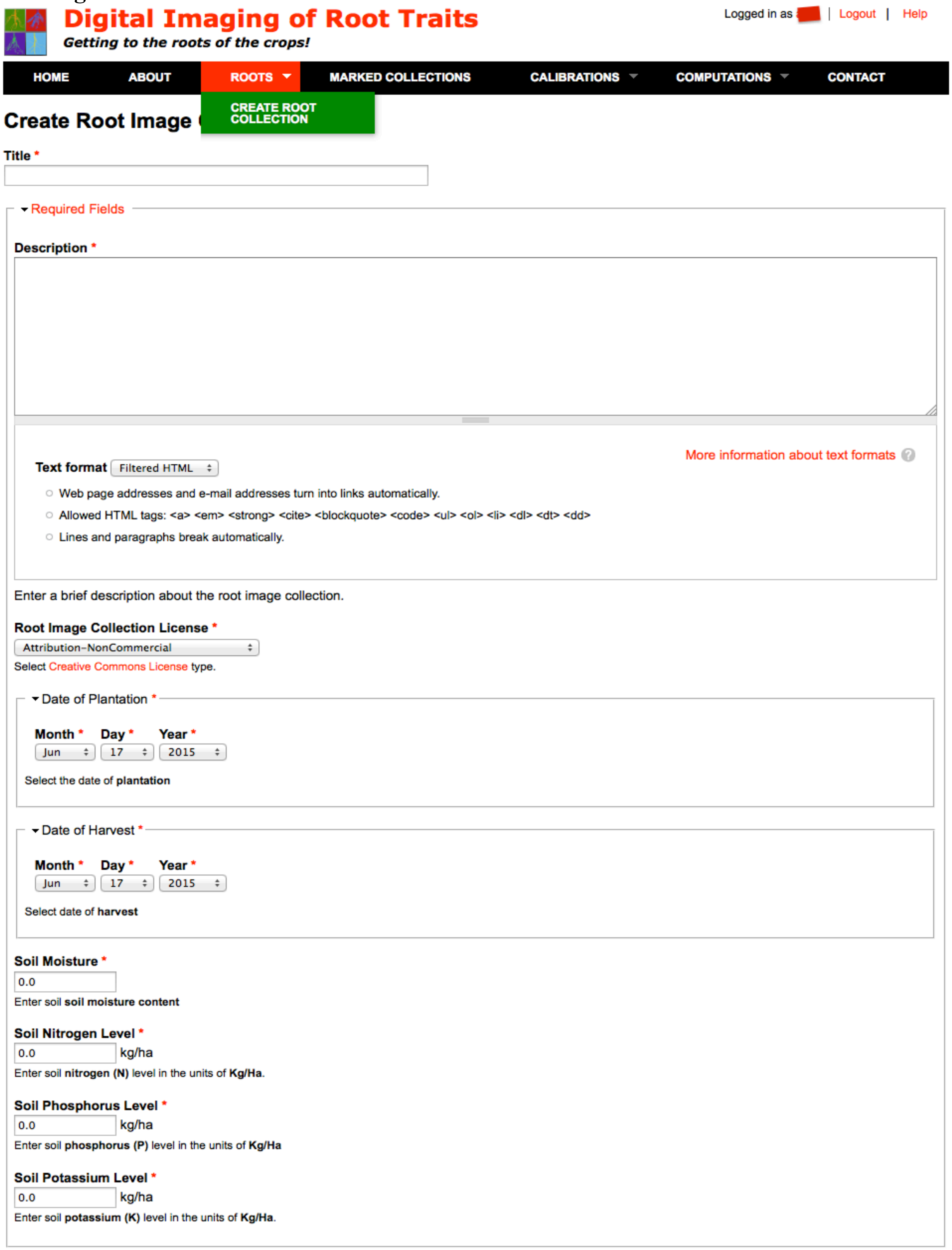

Figure S5: Screenshot of the DIRT's user interface to create a root image collection. It shows a portion (i.e. required field's) of the entire interface. All field names shown here are self-explanatory. All fields have default values; with the exception of 'Title' and 'Description' fields, edit them as appropriate for the collection.

9.6 Figure S6

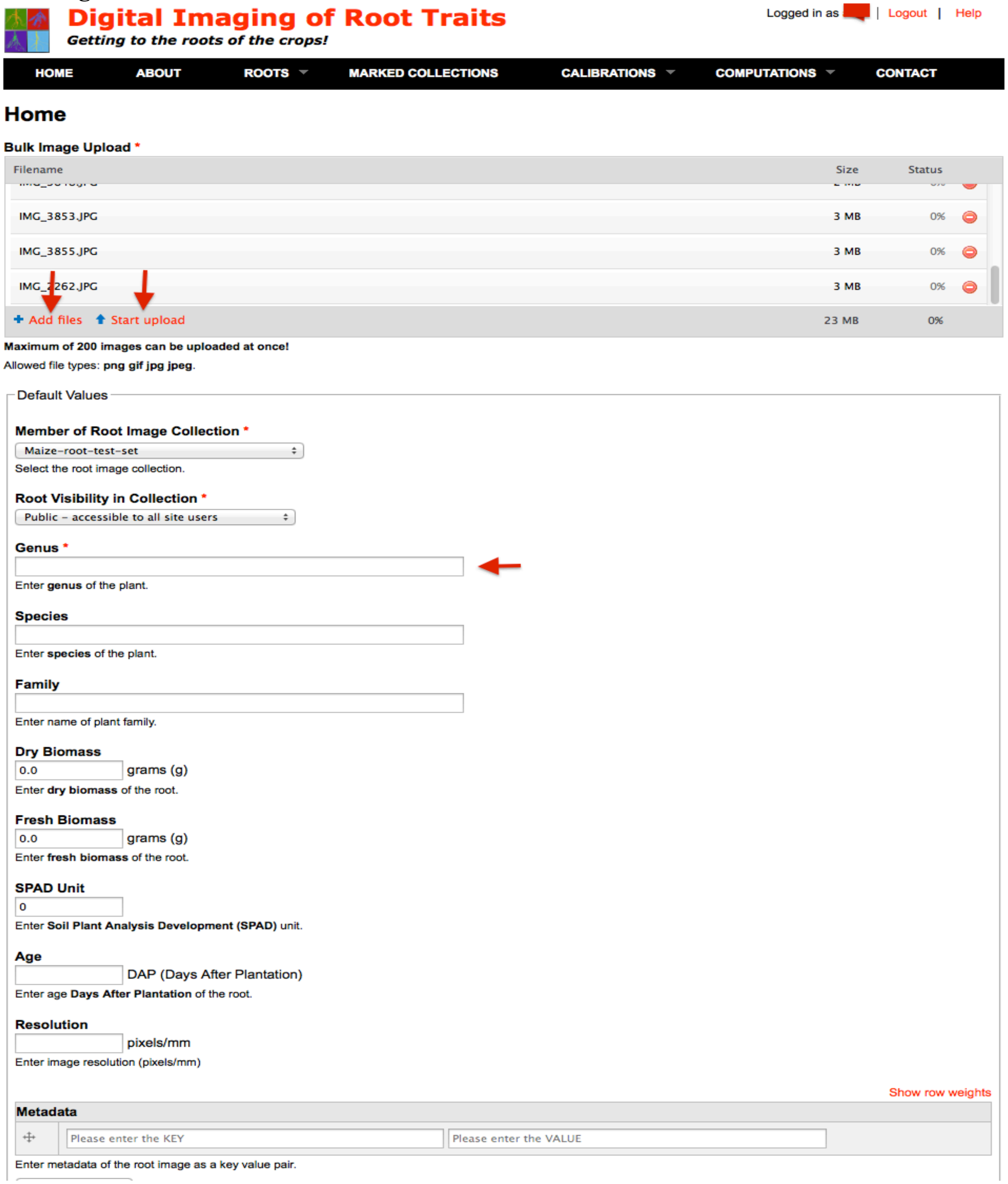

Figure S6: Screenshot of the DIRT's user interface to upload and add images to a newly created or an existing collection. It shows a portion of the whole interface. This interface shows metadata fields associated to each image being uploaded to the collection. The red arrows indicate the links and fields that the use has to click and fill in respectively.

9.7 Figure S7

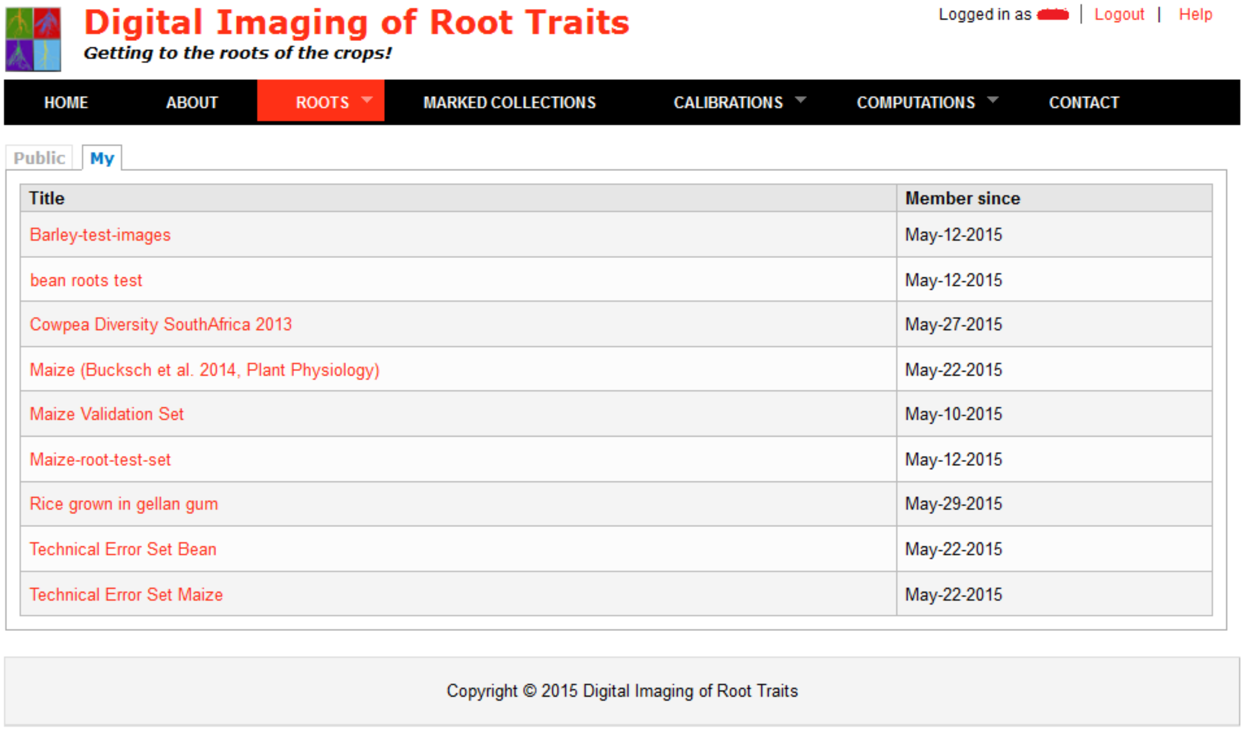

Title	Member since
Barley-test-images	May-12-2015
bean roots test	May-12-2015
Cowpea Diversity SouthAfrica 2013	May-27-2015
Maize (Bucksch et al. 2014, Plant Physiology)	May-22-2015
Maize Validation Set	May-10-2015
Maize-root-test-set	May-12-2015
Rice grown in gellan gum	May-29-2015
Technical Error Set Bean	May-22-2015
Technical Error Set Maize	May-22-2015

Figure S7: Screenshot of the DIRT's user interface that shows the list of root image collections managed by the logged in user.

9.8 Figure S8

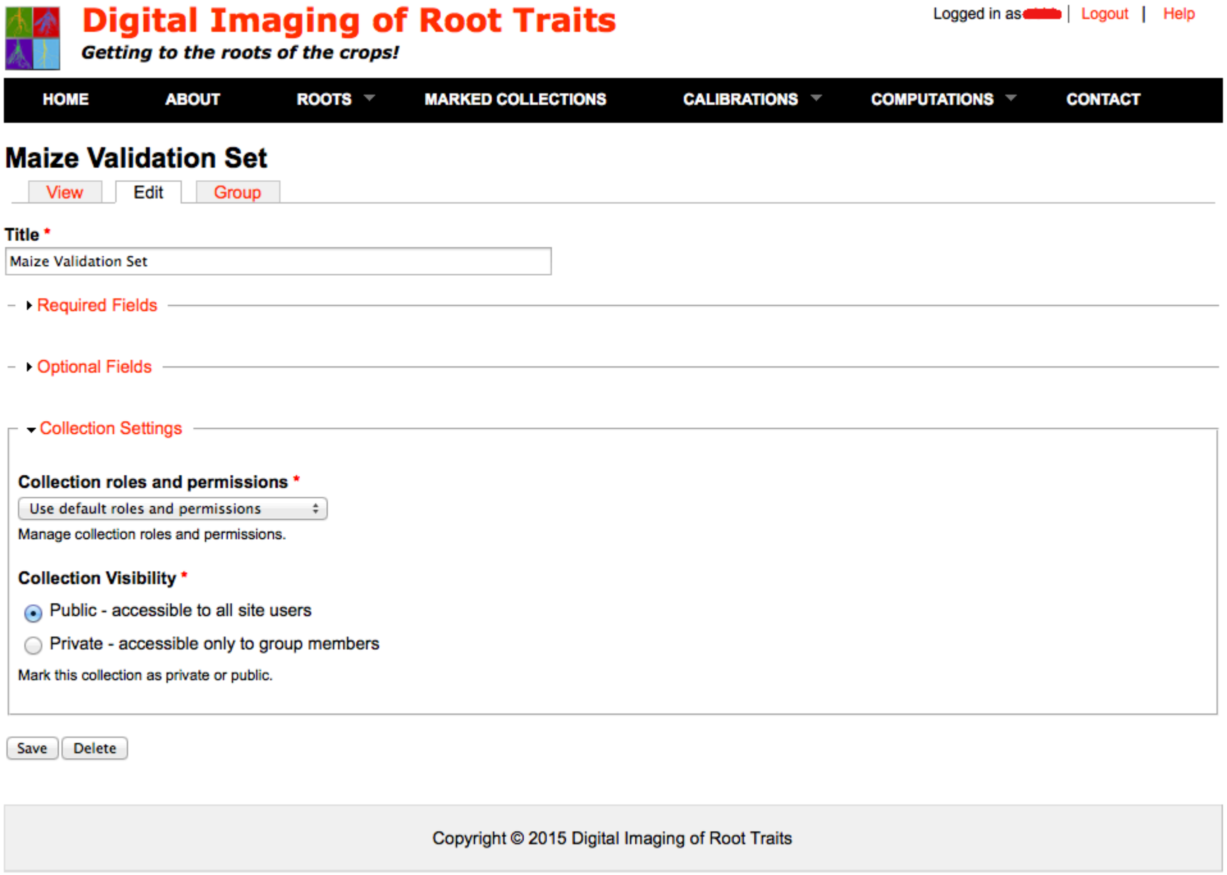

Figure S8: Screenshot of the DIRT's user interface to edit a root image collection and change its collection setting to 'Public'.

9.9 Figure S9

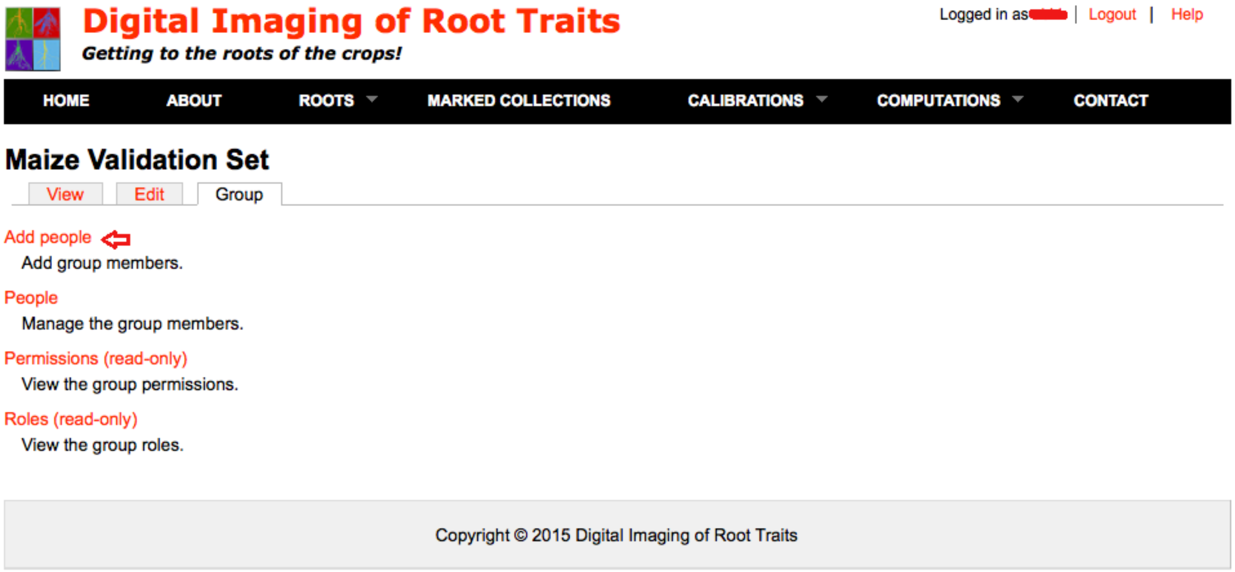

Figure S9: Screenshot of the DIRT's user interface that enables to add and manage members of a root image collection. The red arrow indicated the link the user has to click in order add people/member to the collection.

9.10 Figure S10

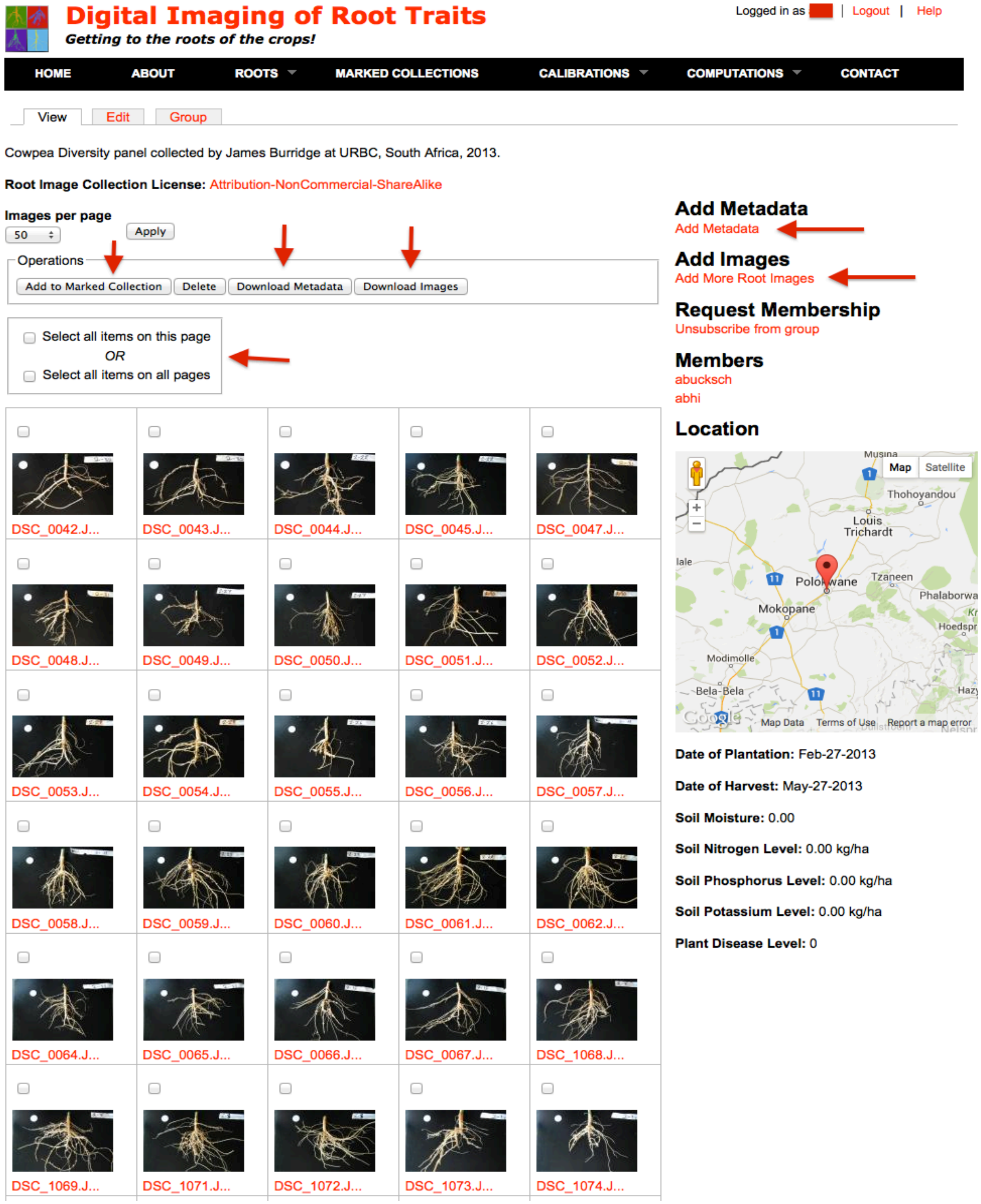

Figure S10: Screenshot of the DIRT's root image collection view interface. A user can perform all functions associated to a collection from this interface. Functions are (a) Edit collection metadata by clicking on the 'Edit' tab, (b) Add/manage people or member of the collection by clicking on the 'Group' tab, (c) Add images to a 'Marked Collection' (section 6.4), (d) Delete selected image(s), (e) Download image metadata, (f) Download images, (g) Add image metadata and (h) Add more root images to the collection. The red arrow indicates the major operations/actions that can be performed on a collection.

9.11 Figure S11

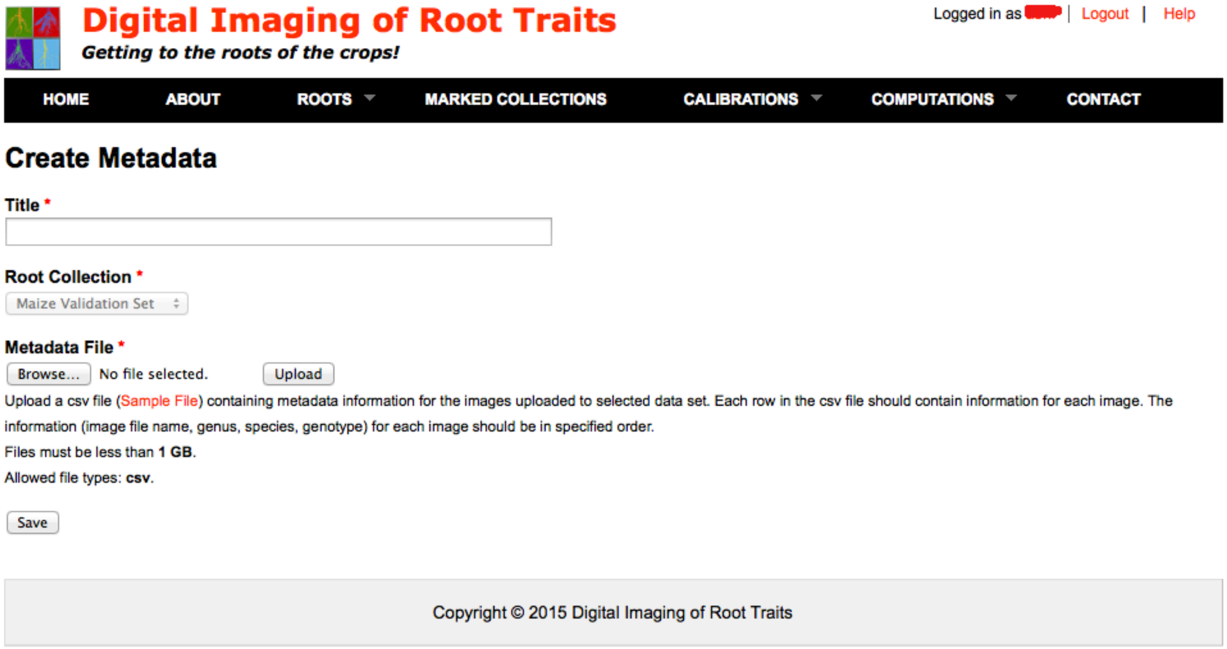

Figure S11: Screenshot of the DIRT's interface to add/update metadata of images in a collection.

9.12 Figure S12

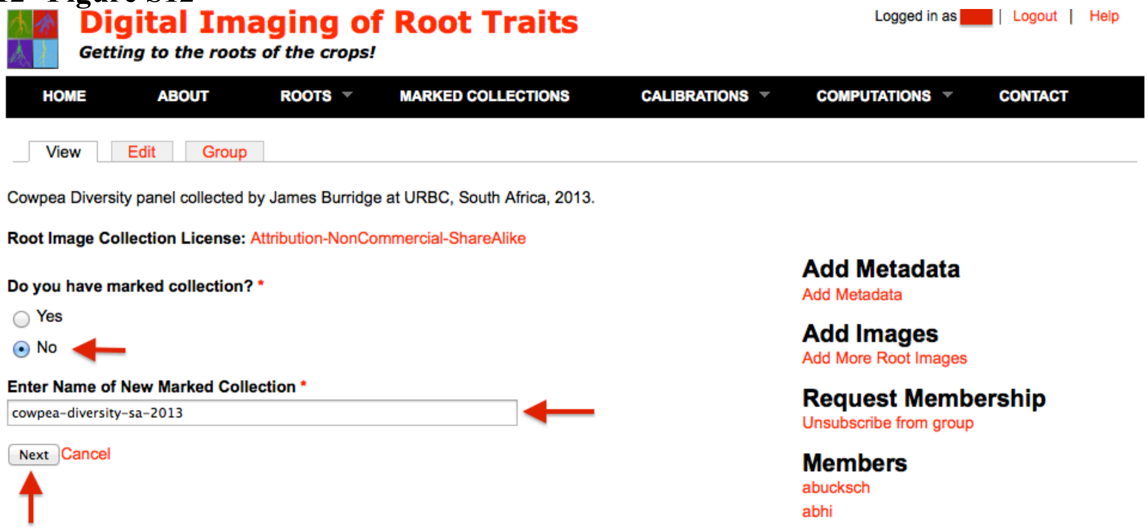

Figure S12: Screenshot of the DIRT's interface to create a new marked collection and add images to it. The red arrow indicates the fields that need user's attention.

9.13 Figure S13

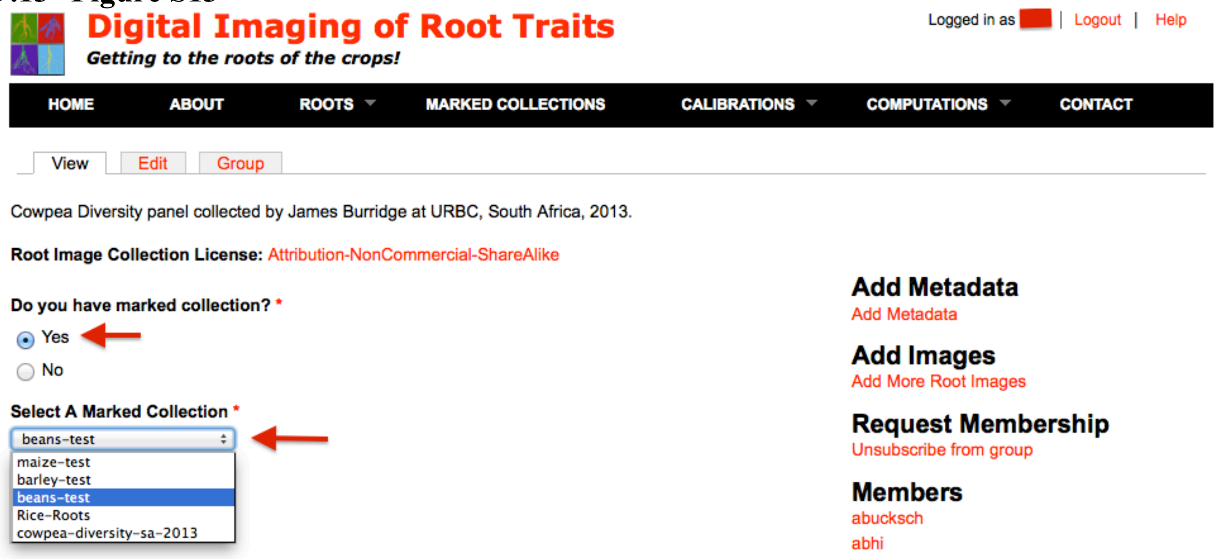

Figure S13: Screenshot of the DIRT's interface to add images to an existing marked collection. The red arrow indicates the fields that need user's attention.

9.14 Figure S14

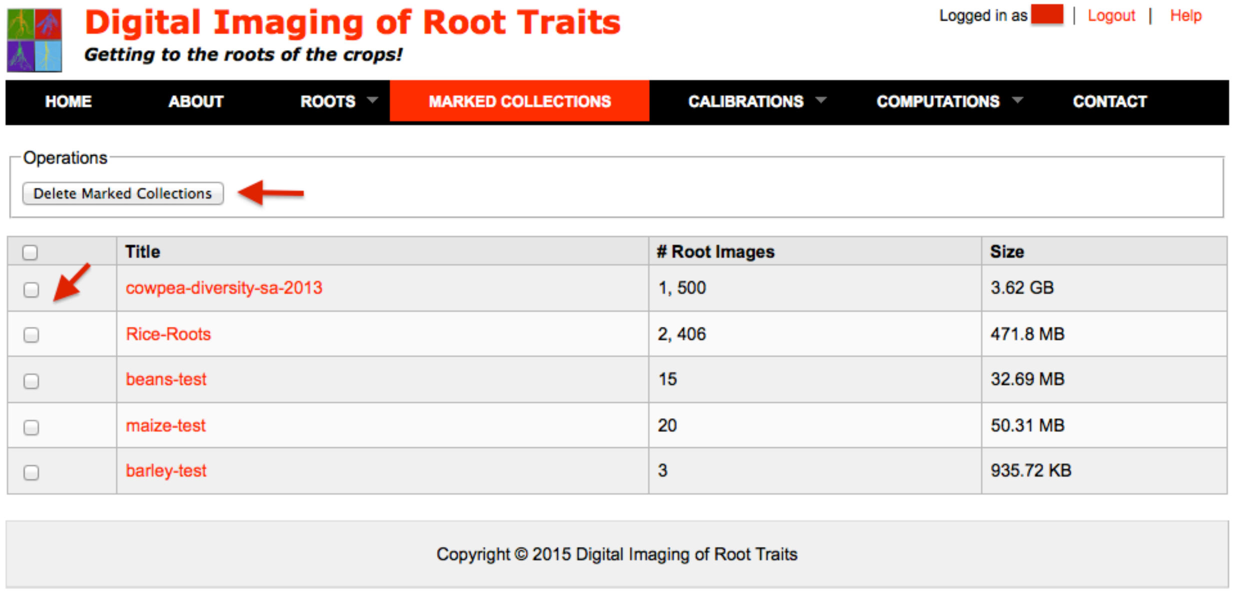

Figure S14: Screenshot of the DIRT's interface that lists the marked collections of the logged in user. The red arrow indicates the fields that need user's attention/action to delete a marked collection.

9.15 Figure S15

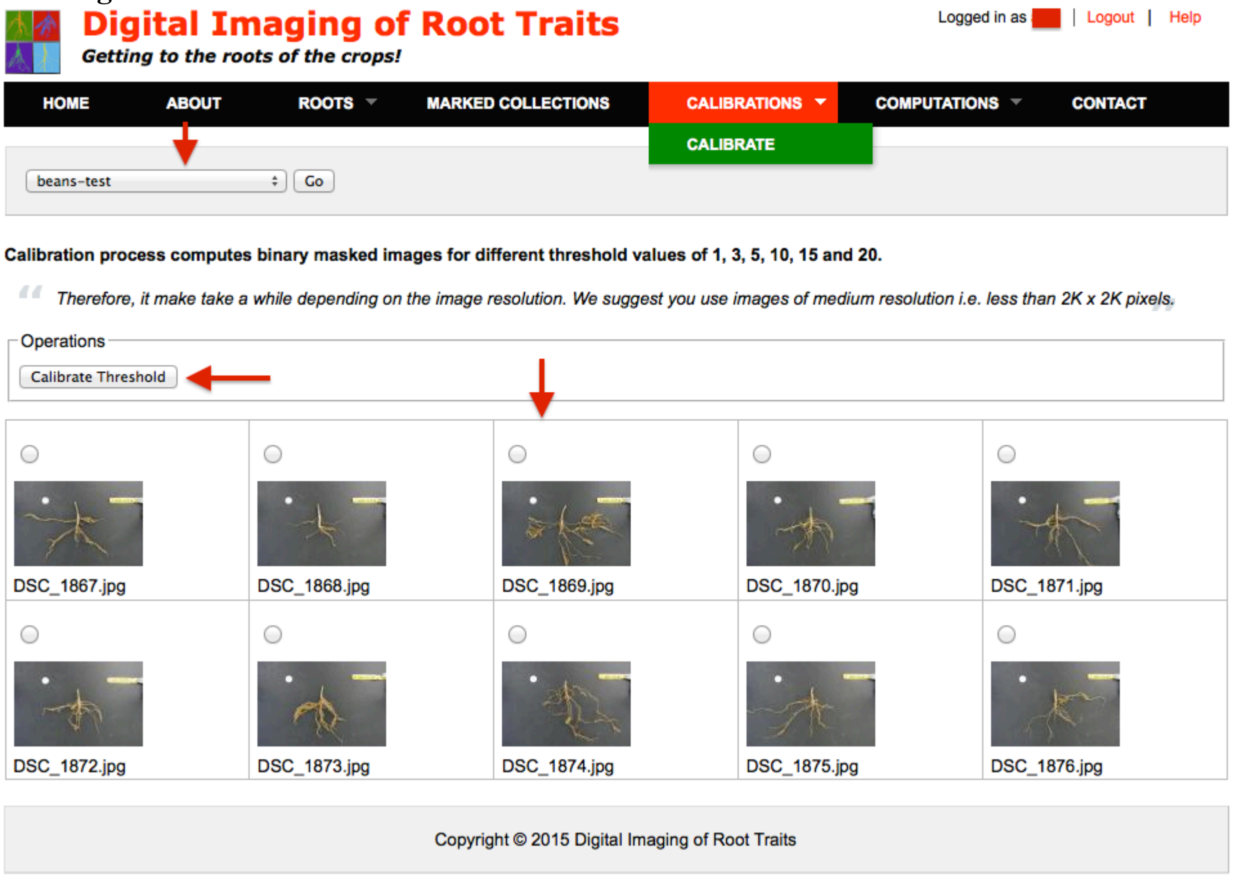

Figure S15: Screenshot of the DIRT's interface to calibrate segmentation threshold value for an image in the marked collection. It works in two steps (a) Select a marked collection from the list as seen in the above figure and click 'Go', this will load the images of the marked collection, and (b) select an image and click on 'Calibrate Threshold' button. The red arrow indicates the fields/buttons that need user's attention/action for threshold calibration.

9.16 Figure S16

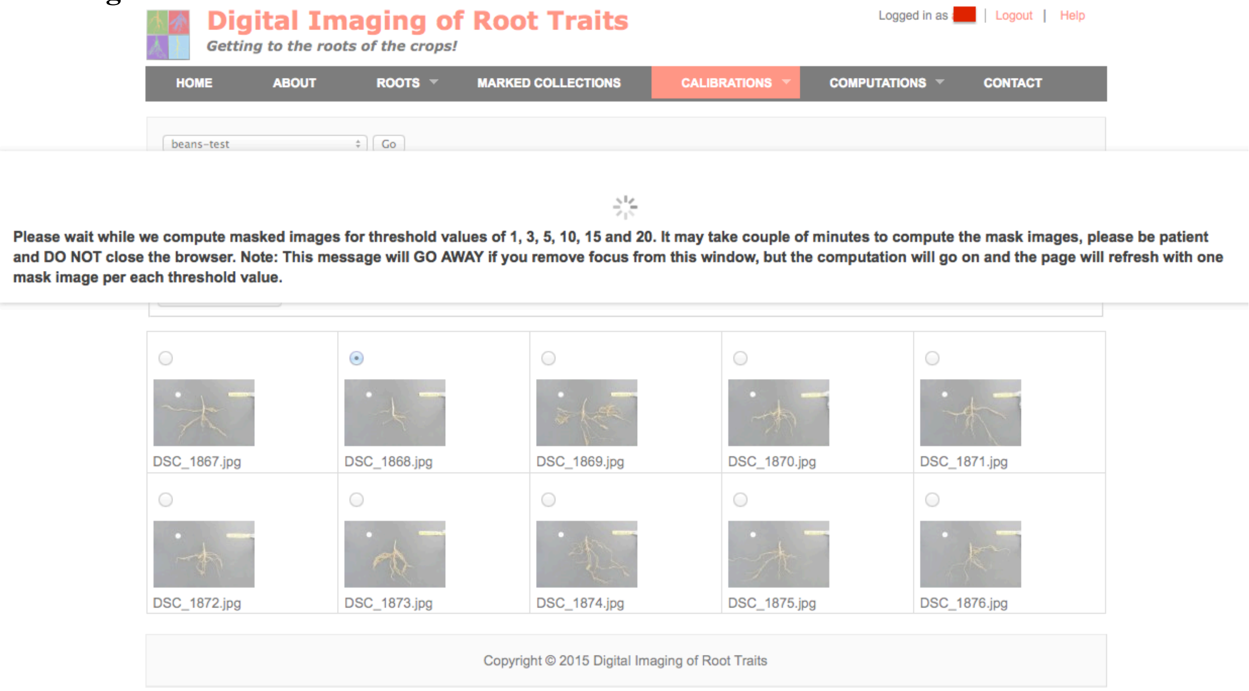

Figure S16: Screenshot of the DIRT's interface that shows up while an image is being calibrated by a user.

9.17 Figure S17

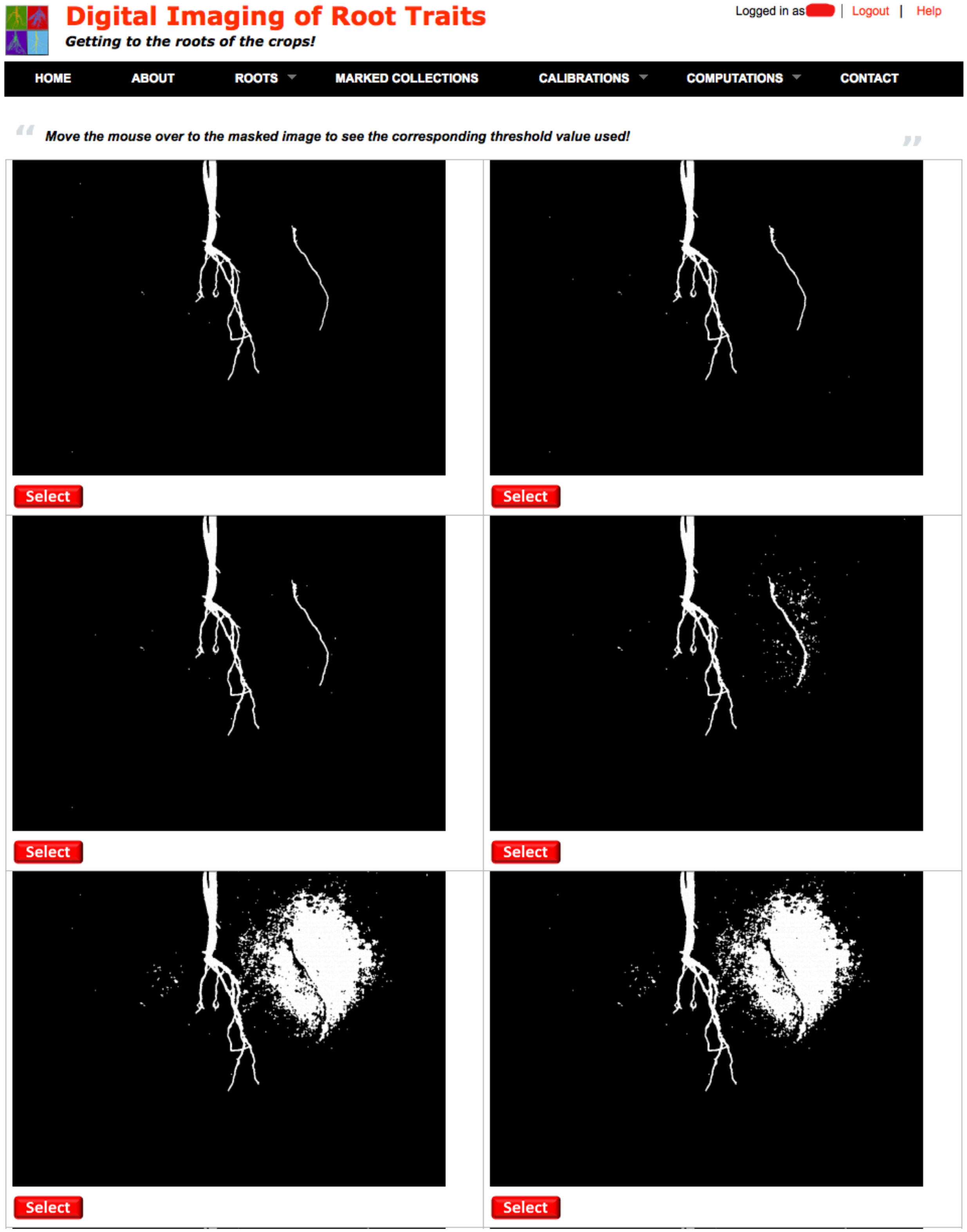

Figure S17: Screenshot of the DIRT's interface that shows up on completion of the calibration. All masked images with threshold values are presented to the user for inspection and selection.

9.18 Figure S18

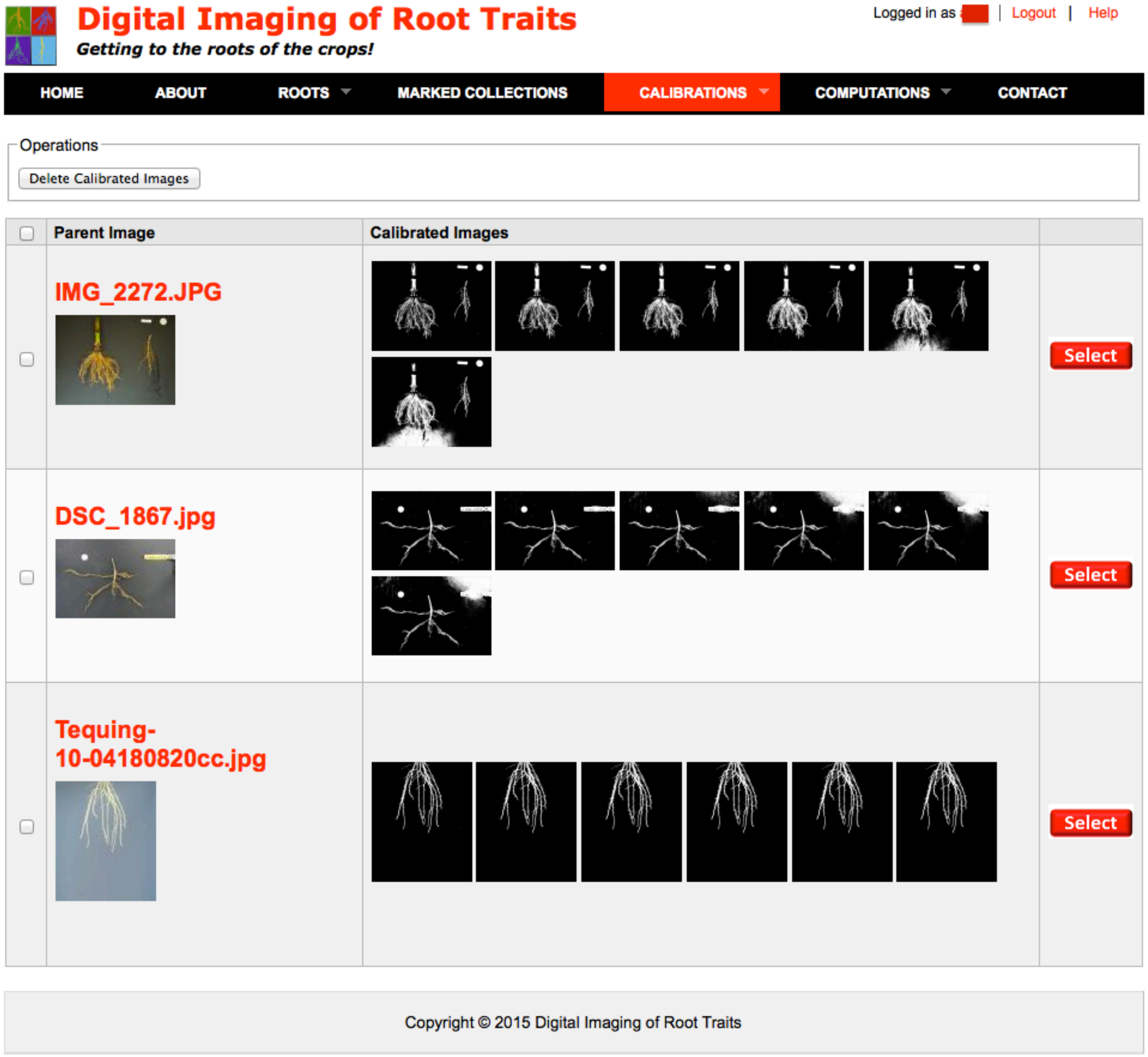

Figure S18: Screenshot of the DIRT's interface that shows the list of calibrations performed by a user.

9.19 Figure S19

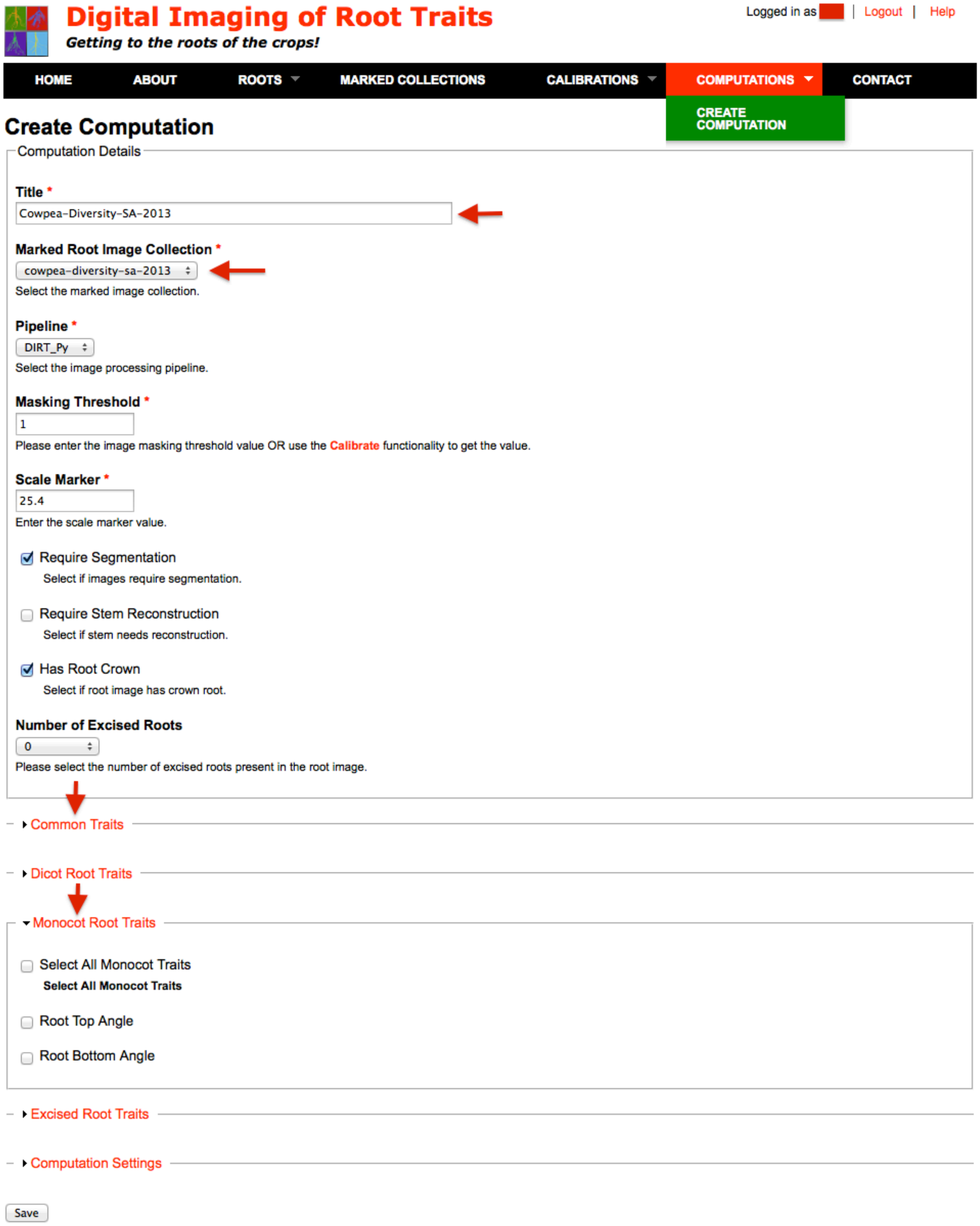

Figure S19: Screenshot of the DIRT's interface to create a new computation. This interface enables the user to give a title to the computation, select the marked collection whose images are to be used for trait computation, select the trait computation pipeline, provide pipeline parameters and select traits of interest. The red arrow indicates the fields that need user's attention.

9.20 Figure S20

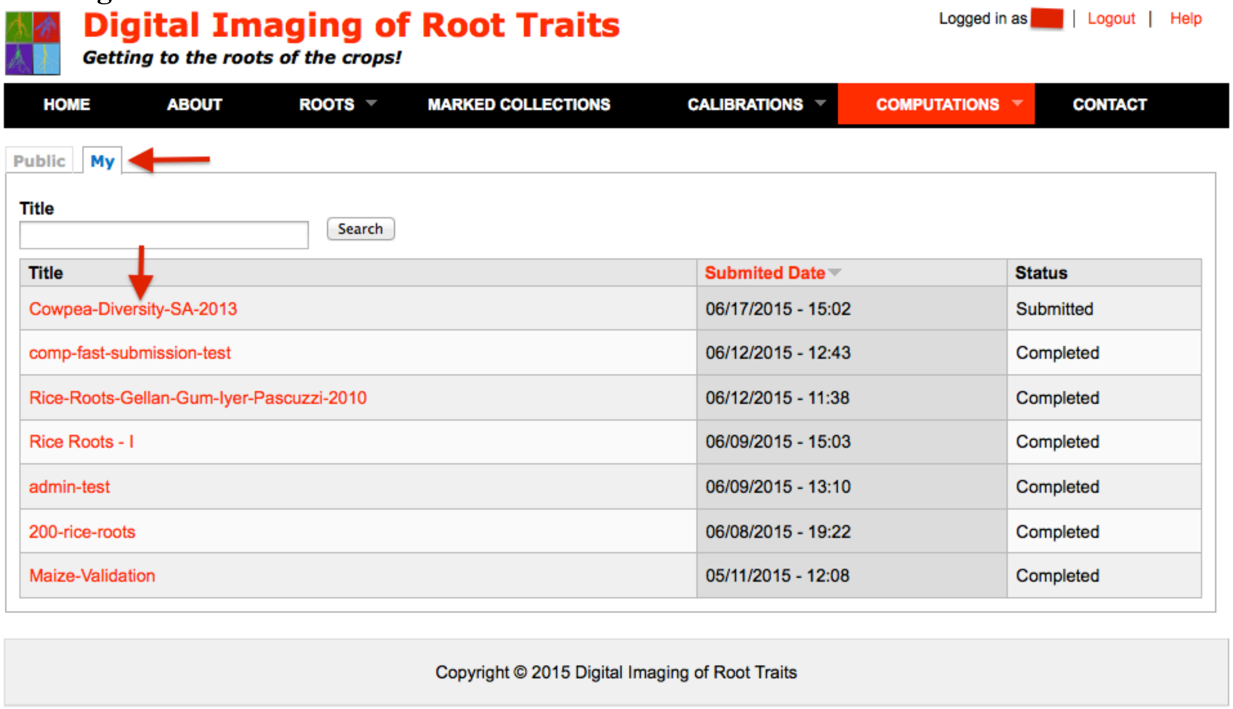

Figure S20: Screenshot of the DIRT's interface that shows the list of computations managed/associated to the logged in user. The red arrow indicates the fields that need user's attention/action.

9.21 Figure S21

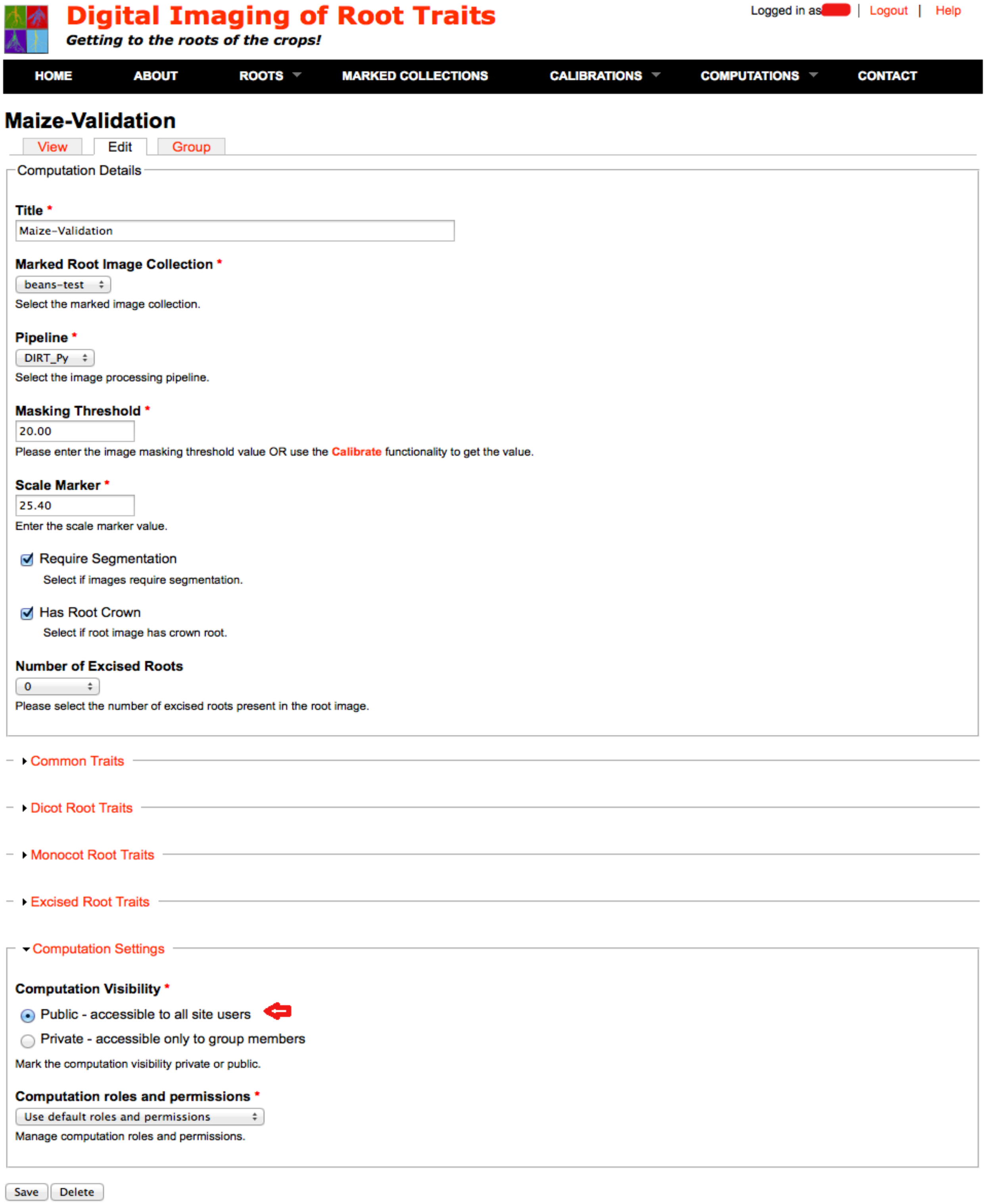

Figure S21: Screenshot of the DIRT's interface that enables user to start/create a RSA trait computation process on the underlying high-throughput grid computing environment. The red arrow indicates the fields that need user's attention/action.

9.22 Figure S22

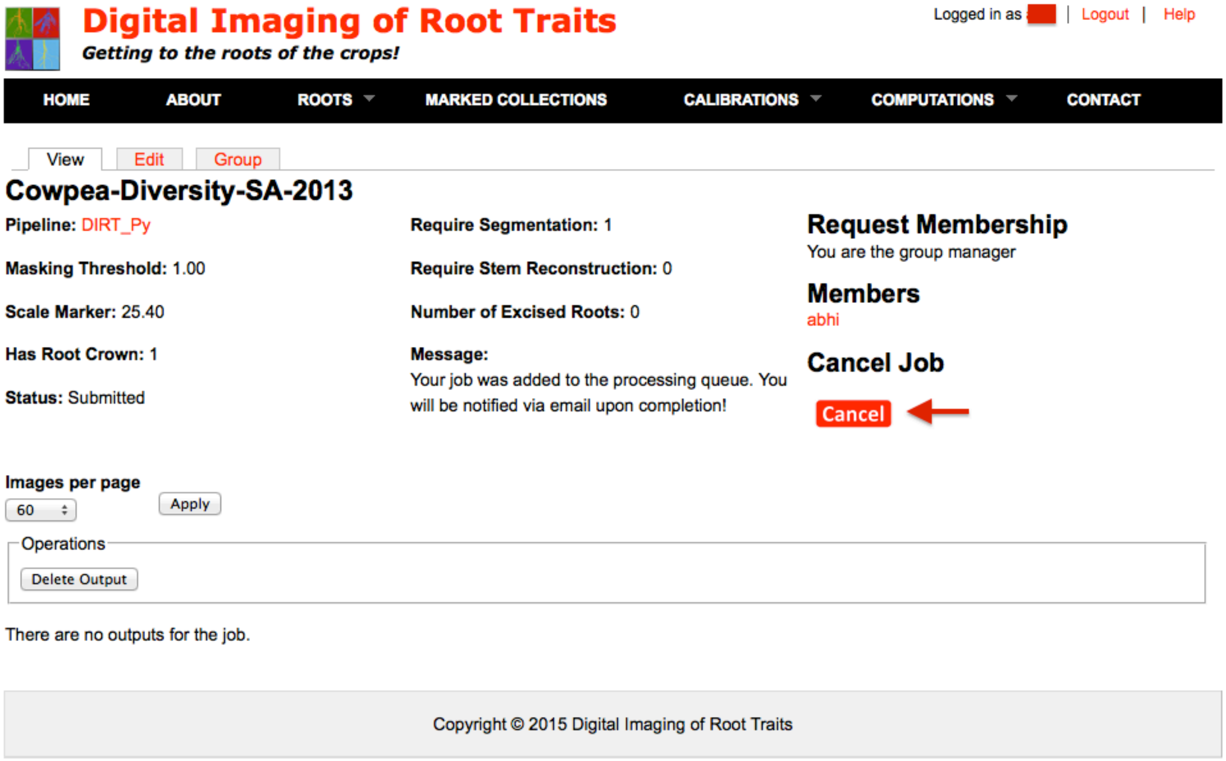

Figure S22: Screenshot of the DIRT's interface that allows the user to cancel a submitted or running computation. The red arrow indicates the button that need user's action to cancel a computation.

9.23 Figure S23

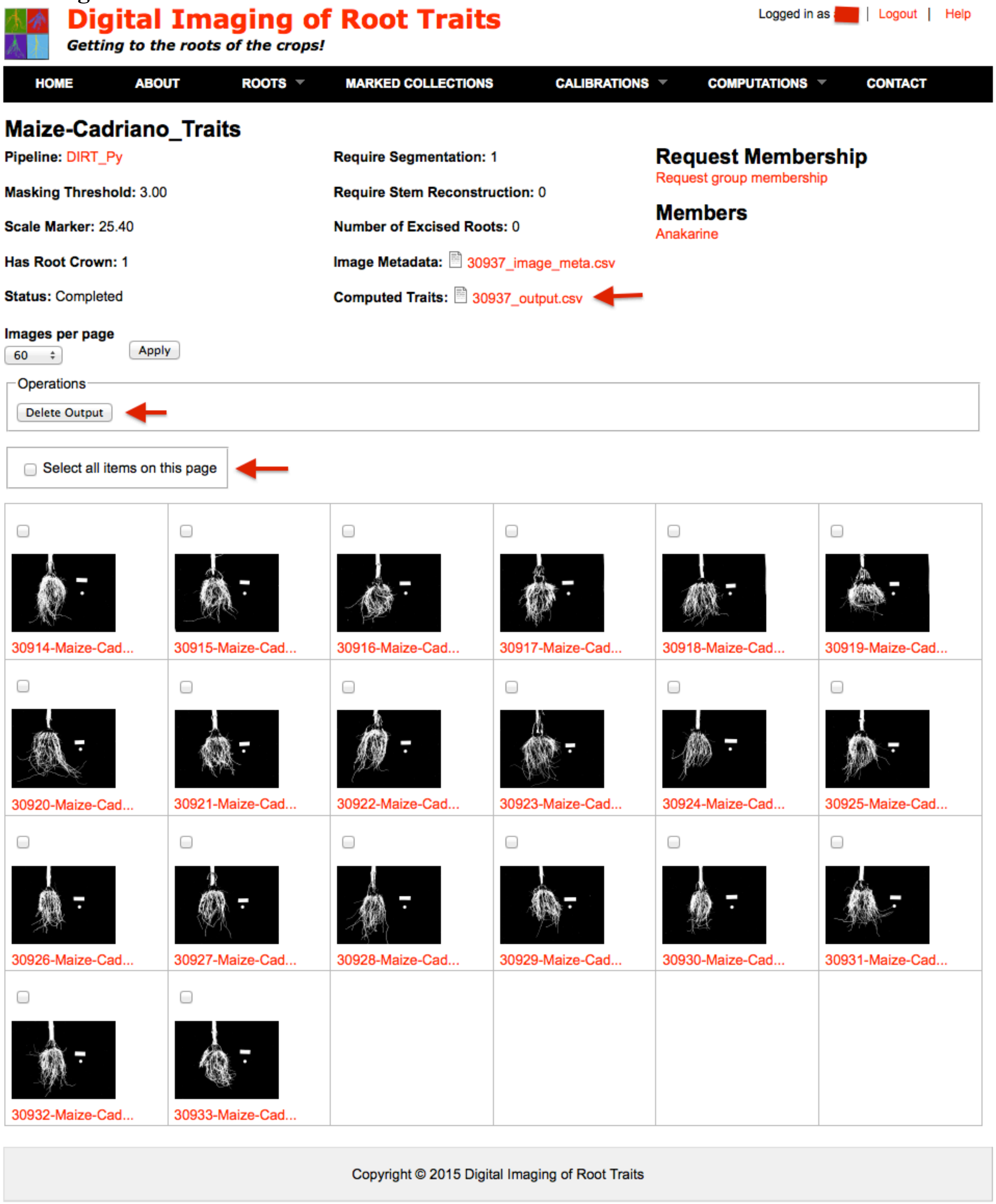

Figure S23: Screenshot of the DIRT's interface of a completed computation. The user can visually inspect the masked images and download the computed traits and metadata. The red arrow indicates the fields that need user's attention/action.

9.24 Figure S24

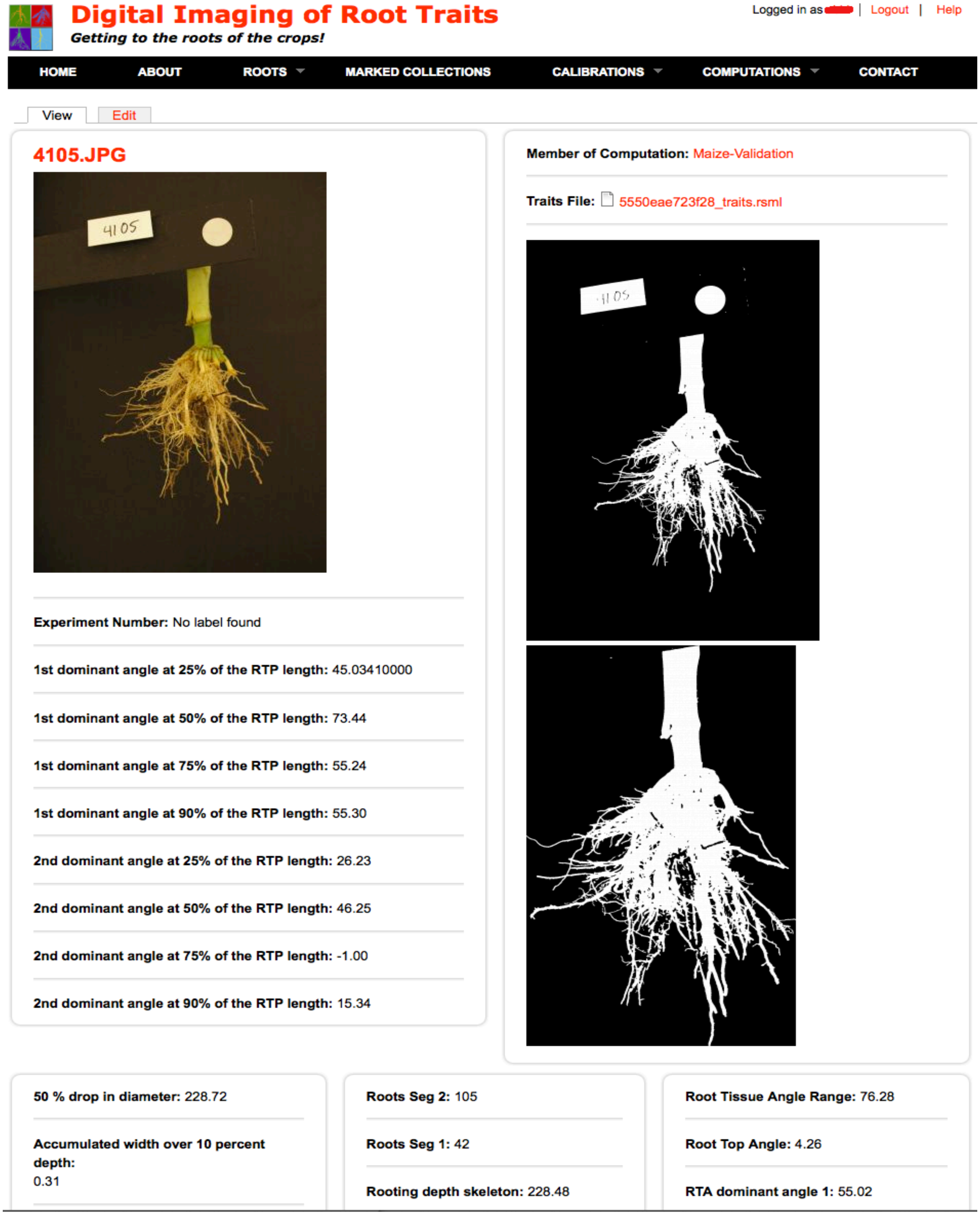

Figure S24: Screenshot of the DIRT's interface showing computed traits of a single image. The computed traits are available for download in the form of a RSML file. Note, this figure is not showing all the traits, but all the computed traits can actually be seen on the site by scrolling down the page.

9.25 Figure S25

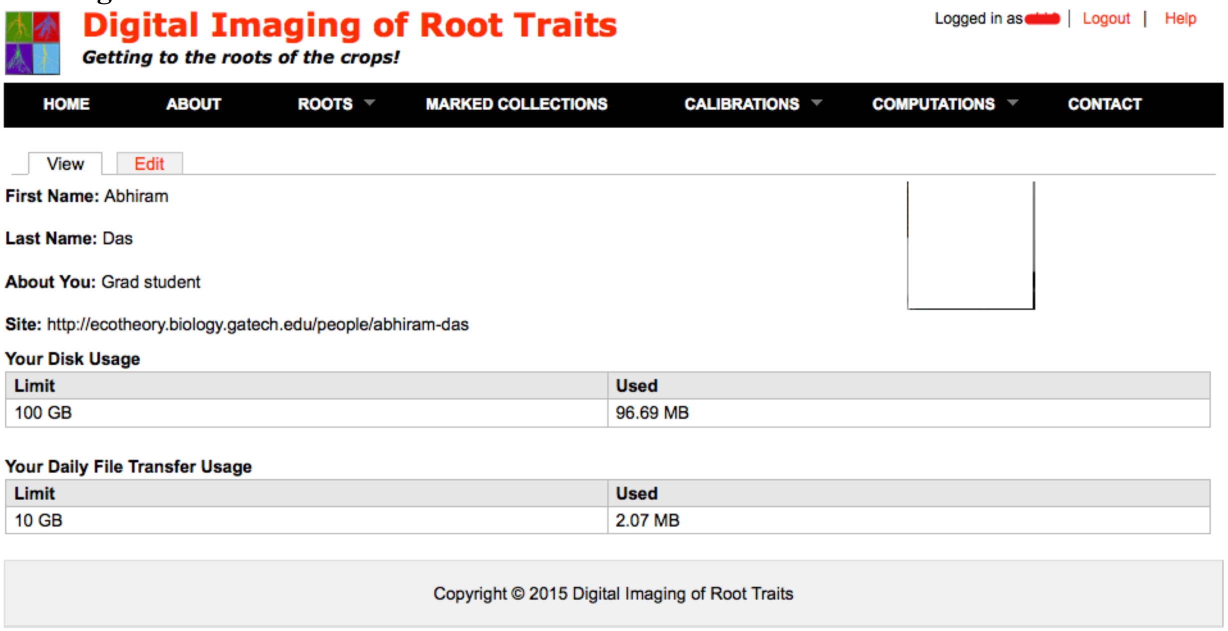

Figure S25: Screenshot of the DIRT's interface showing user profile details.

9.26 Figure S26

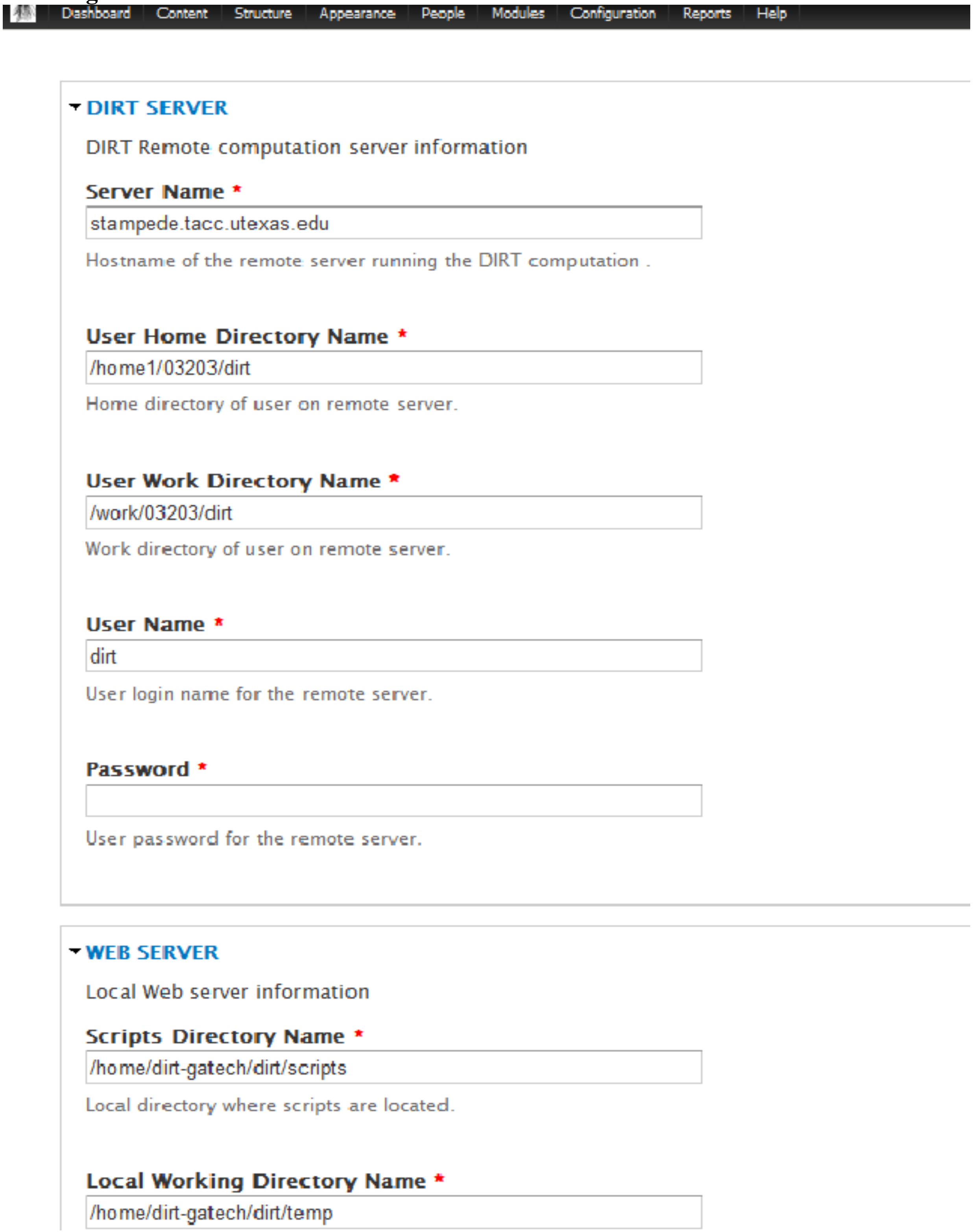

Figure S26: Screenshot of the DIRT's administration interface to configure grid computing platform information.

9.27 Figure S27

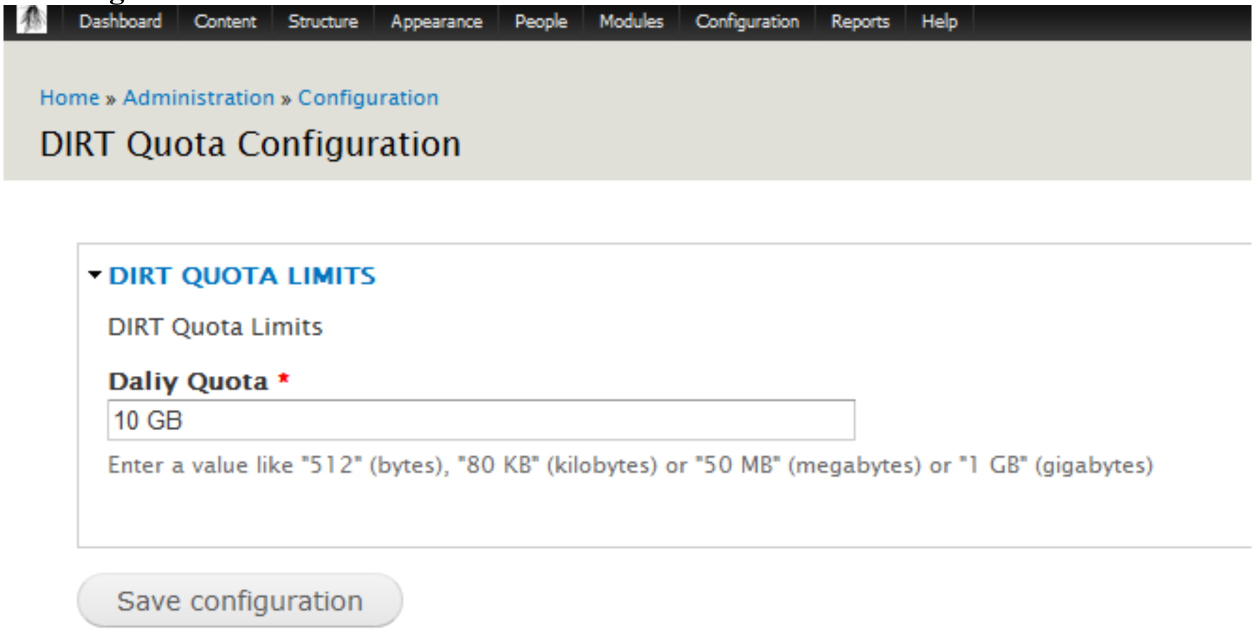

Figure S27: Screenshot of the DIRT's administration interface to configure user's daily computation quota information.

9.28 Figure S28

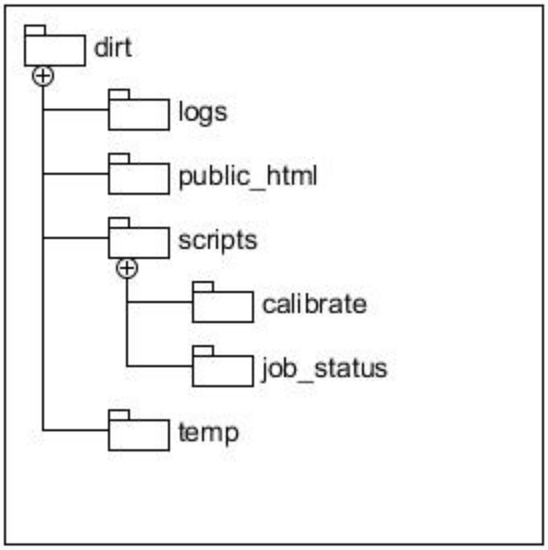

Figure S28: Directory structure on DIRT web server node

9.29 Figure S29

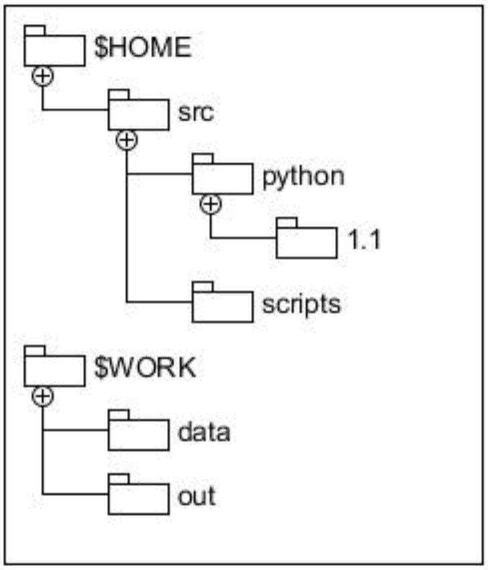

Figure S29: Directory structure on DIRT grid node

10 References

[1] A. Bucksch, J. Burridge, L. M. York, A. Das, E. Nord, J. S. Weitz, and J. P. Lynch, "Image-Based High-Throughput Field Phenotyping of Crop Roots," *Plant Physiology,* vol. 166, no. 2, pp. 470-486, Oct, 2014.

[2] T. Colombi, N. Kirchgessner, C. A. Le Marie, L. M. York, J. P. Lynch, and A. Hund, "Next generation shovelomics: set up a tent and REST," *Plant and Soil,* vol. 388, no. 1-2, pp. 1-20, Mar, 2015.

[3] T. Galkovskyi, Y. Mileyko, A. Bucksch, B. Moore, O. Symonova, C. A. Price, C. N. Topp, A. S. Iyer-Pascuzzi, P. R. Zurek, S. Q. Fang, J. Harer, P. N. Benfey, and J. S. Weitz, "GiA Roots: software for the high throughput analysis of plant root system architecture," *Bmc Plant Biology,* vol. 12, Jul 26, 2012.

[4] M. A. Fischler, and R. C. Bolles, "Random Sample Consensus - a Paradigm for Model-Fitting with Applications to Image-Analysis and Automated Cartography," *Communications of the Acm,* vol. 24, no. 6, pp. 381-395, 1981.

[5] G. Lobet, M. P. Pound, J. Diener, C. Pradal, X. Draye, C. Godin, M. Javaux, D. Leitner, F. Meunier, P. Nacry, T. P. Pridmore, and A. Schnepf, "Root System Markup Language: Toward a Unified Root Architecture Description Language," *Plant Physiology,* vol. 167, no. 3, pp. 617-627, Mar, 2015.

[6] A. Bucksch, "A Practical Introduction to Skeletons for the Plant Sciences," *Applications in Plant Sciences,* vol. 2, no. 8, Aug, 2014.

[7] A. S. Iyer-Pascuzzi, O. Symonova, Y. Mileyko, Y. L. Hao, H. Belcher, J. Harer, J. S. Weitz, and P. N. Benfey, "Imaging and Analysis Platform for Automatic Phenotyping and Trait Ranking of Plant Root Systems," *Plant Physiology,* vol. 152, no. 3, pp. 1148-1157, Mar, 2010.

[8] Y. L. Chen, V. M. Dunbabin, J. A. Postma, A. J. Diggle, J. A. Palta, J. P. Lynch, K. H. M. Siddique, and Z. Rengel, "Phenotypic variability and modelling of root structure of wild Lupinus angustifolius genotypes," *Plant and Soil,* vol. 348, no. 1-2, pp. 345-364, Nov, 2011.

[9] "Drupal, https://www.drupal.org/ [Accessed 16 June 2015]."

[10] "STAMPEDE at TACC. https://www.tacc.utexas.edu/systems/stampede [Accessed 16 June 2015]."
